# Supplementary material for: Ice-Age Climate Adaptations Trap the Alpine Marmot in a State of Low Genetic Diversity
Source: Curr Biol. 2019 May 20;29(10):1712–1720.e7. doi: 10.1016/j.cub.2019.04.020 (PMC6538971; doi:10.1016/j.cub.2019.04.020)
Supplement: Document S1. Figures S1–S4 and Tables S1–S3 [file mmc1.pdf]

Current Biology, Volume *29*

Supplemental Information

Ice-Age Climate Adaptations Trap the Alpine Marmot in a State of Low Genetic Diversity

Toni I. Gossmann, Achchuthan Shanmugasundram, Stefan Börno, Ludovic Duvaux, Christophe Lemaire, Heiner Kuhl, Sven Klages, Lee D. Roberts, Sophia Schade, Johanna M. Gostner, Falk Hildebrand, Jakob Vowinckel, Coraline Bichet, Michael Mülleder, Enrica Calvani, Aleksej Zelezniak, Julian L. Griffin, Peer Bork, Dominique Allaine, Aurélie Cohas, John J. Welch, Bernd Timmermann, and Markus Ralser

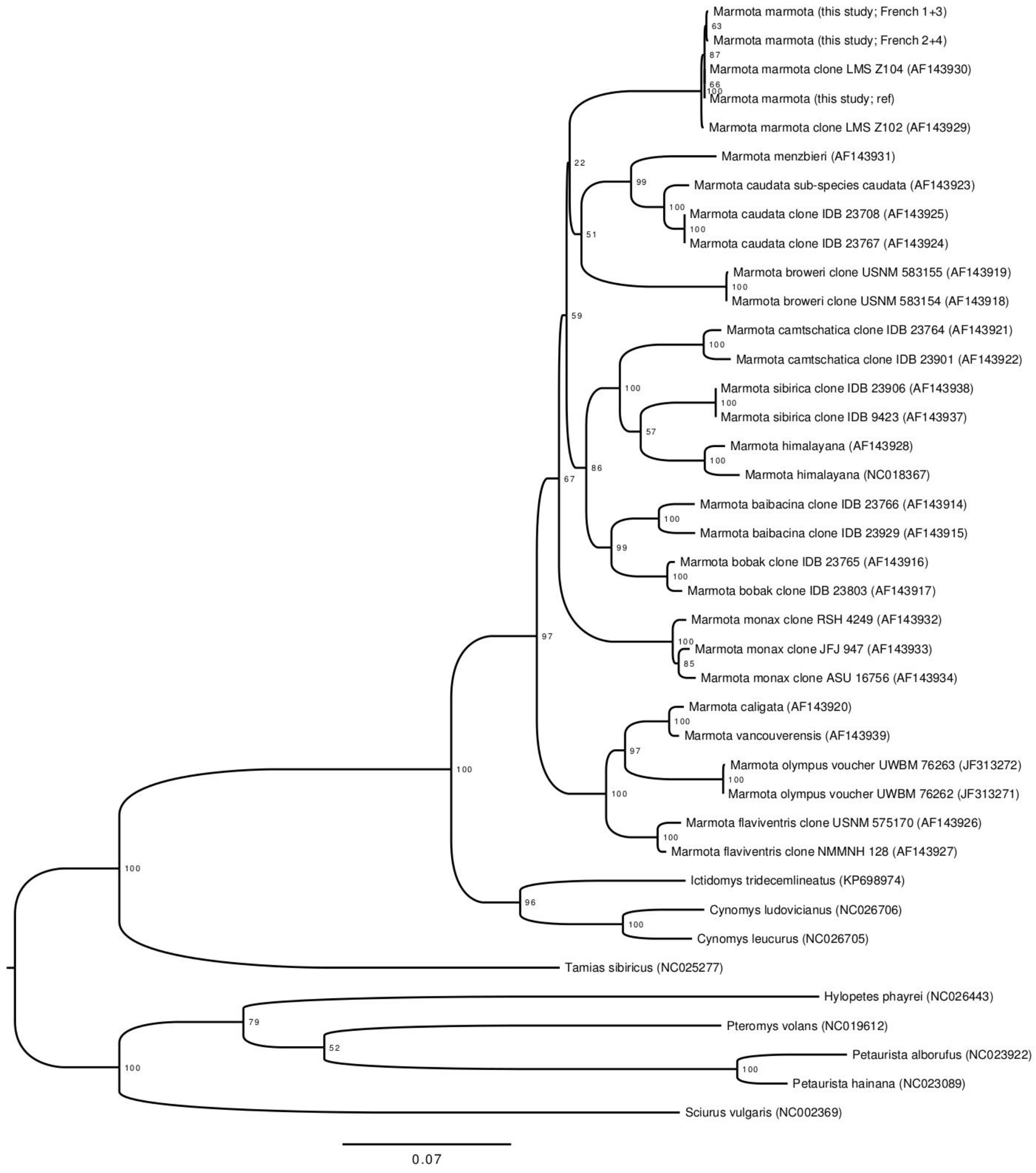

Figure S1. Phylogeny of the marmotini CytB gene confirms the phylogenetic position of the Alpine marmot. Related to Figure 1. We analysed the available sequences of Cytochrome B gene from the subfamilies Xerinae and Sciurinae, after combining these data with homologous sequences obtained from [S1]. The three distinct *CytB* sequences we sampled grouped with existing Alpine marmot specimens with 100% bootstrap support.

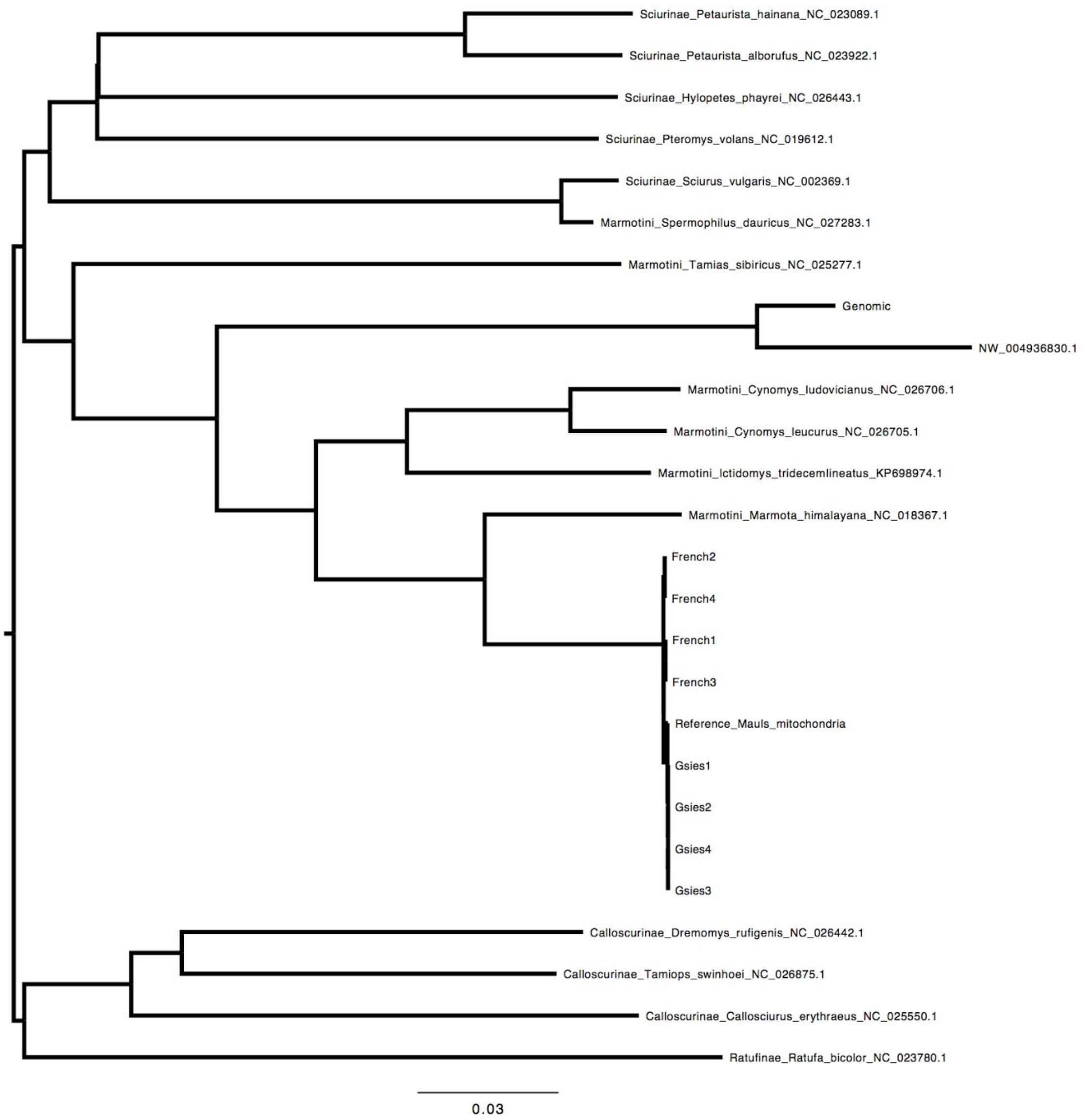

Figure S2. Phylogenetic tree based on mitochondrial genomes for multiple *Marmotini*, and sections of from the nuclear genome comprising an integration of the complete mitochondrial genome (an NUMT event). Related to Figure 1. The tree includes mitochondrial genomes of all resequenced individuals from Alpine marmots, reference mitochondrial genomes available for other *Marmotini* species in public database. The NUMT sequences from Alpine marmot is labelled '*Genomic*' in the tree, and and equivalent sequence from the nuclear genome of the thirteen-lined ground squirrel is labelled as '*NW_004936830.1*'. The placement of these sequences suggests that the NUMT was present in the common ancestor of all *Marmota*, *Ictidomys* and *Cynomys* species, but occured after their common ancestor with the Siberian chipmunk (*Tamias sibiricus*). In other words, the nuclear integration of the mitochondrial genome occurred after the divergence of *Xerinae* (Figure 1C) from other subfamilies of *Sciuridae*. The abbreviation 'French' refers to the La Grande Sassière sample 1-4.

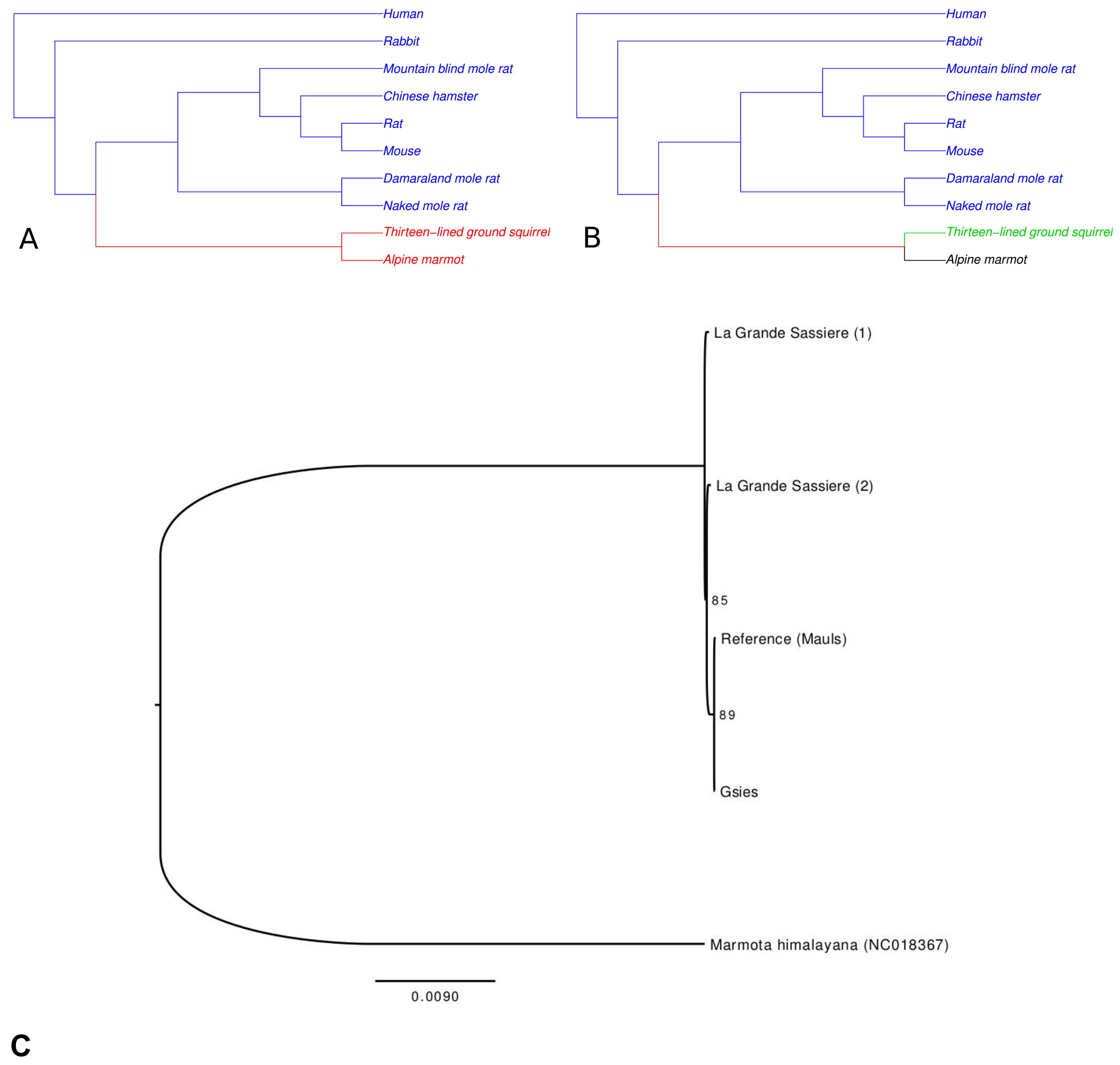

Figure S3. Underlying phylogenetic relationship of the genomes used for substitution rate analyses in PAML of protein coding genes and a genealogy from the mitochondrial genomes of our sample of sequenced Alpine marmots, rooted with the published mitochondrial genome of the Himalayan marmot (*Marmota himalayana, [S2]*). Related to Figures 2,3. Two different branch models have been used, the colours denote the branch classifications for the applied branch-site tests in PAML. (A) A two branch model and (B) a four branch model. Note, that the PAML model treats the tree as unrooted. (C) Node labels show bootstrap support, and branch lengths are shown in units of substitution per site. This genealogy supports our contention in the main text that the diversity of the two subpopulations with lower effect size (Mauls and Gsies) nests within the diversity of the subpopulation with larger effect size, from La Grande Sassiere (LGS). This is equivalent to saying that the most recent common ancestor of sequences in the LGS population is also a direct ancestor of all sequences in the sample. In particular, a grouping of the Mauls and Gsies sequences and one of the LGS sequences, to the exclusion of the other, has 85% bootstrap support. This alignment excludes any site with a gap, and support for the grouping increases to 89% when gaps are included (not shown).

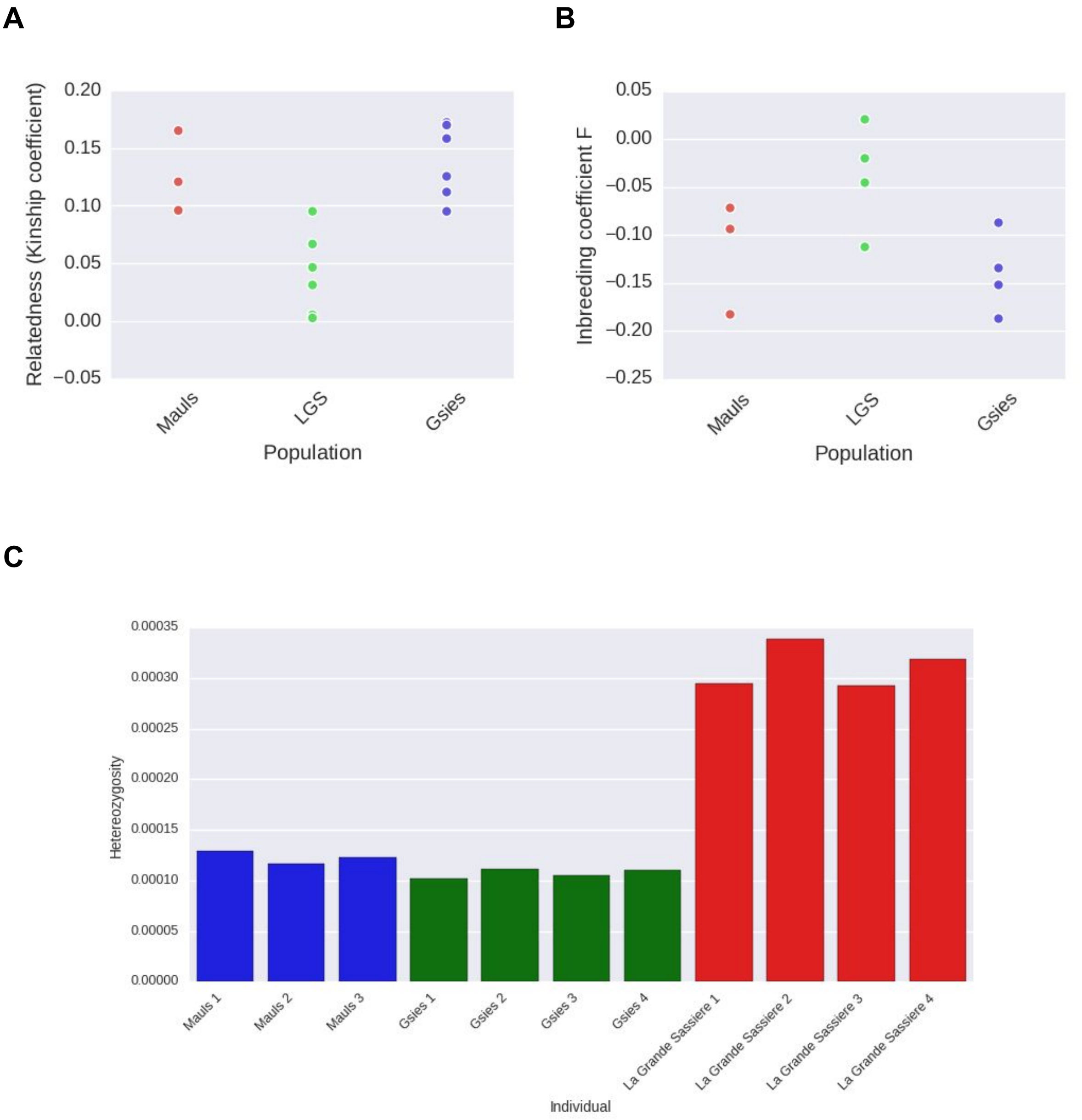

Figure S4. Population specific estimates of relatedness, inbreeding coefficients and genomic diversity. Related to Figure 3. (A) Relatedness coefficients [S3] calculated for each pair of individuals per population (3 individuals for Mauls, 4 for Gsies and 4 for LGS). A kinship coefficient of 0 denotes unrelated individuals, while a kinship coefficient of 0.5 a monozygotic twin. Individuals from Mauls and Gsies show higher levels of relatedness, similar to 2nd-degree kinship, while individuals from LGS are largely unrelated. (B) Inbreeding coefficients F, calculated for each individual within the respective subpopulation. (C) Genome-level nucleotide heterozygosity in the Alpine marmot cohorts from three different populations, Mauls (excluding the reference genome), Gsies (I), and La Grande Sassière (F). Average heterozygosity per individual is shown. Mauls and Gsies populations exhibit similar heterozygosity, whereas the animals of La Grande Sassière are around twice as diverse.

Higher in Alpine Marmot WAT		Higher in rat and mouse WAT	
M/Z	ID	M/Z	ID
738.782	DG 44:8	852.847	TG 50:0
908.913	TG 55:7	829.802	TG 50:3
900.851	TG 55:11	816.843	TG 48:4
892.883	TG 54:8	873.83	TG 53:2
896.912	TG 54:6	824.899	TG 48:0
902.954	TG 54:4	680.695	DG 39:2
864.851	TG 52:8	880.88	TG 52:0
868.874	TG 52:6	895.851	TG 55:5
894.892	TG 54:7	896.91	TG 54:6
892.876	TG 54:8	854.861	TG 51:6
862.92	TG 51:2	880.969	TG 52:0
921.869	TG 58:13	926.862	TG 56:5
872.819	TG 52:4	830.86	TG 49:4
890.953	TG 53:2	798.796	TG 47:6
925.901	TG 58:11	575.611	DG 33:4
911.893	TG 57:11		
884.905	TG 53:5		
896.821	TG 54:6		
858.888	TG 51:4		
861.827	TG 53:8		
848.817	TG 51:9		
886.921	TG 53:4		
876.936	TG 53:9		
904.97	TG 55.9		

840.838	TG 50:6		
890.868	TG 54:9		
898.923	TG 54:5		
882.89	TG 53:6		

Table S1. Enrichment of different triacylglycerol and diacylglycerol lipids, Related to Figure 2. Enrichment of different triacylglycerol and diacylglycerol lipids in Alpine marmot WATs compared to WATs of male Bl/6 mice and Wistar rats.

**Individual**	**Number of RoH > 2MB**	**Percentage of genome with RoH>2MB**
La Grande Sassière1	43	6.37%
La Grande Sassière2	21	2.54%
La Grande Sassière3	48	8.05%
La Grande Sassière4	30	4.14%
Gsies1	159	26.86%
Gsies2	139	21.81%
Gsies3	158	25.41%
Gsies4	147	23.05%
Mauls1	84	12.64%
Mauls2	115	15.81%
Mauls3	135	20.22%

Table S2. **Results of run of homozygosity analyses, Related to Figure 3.** Results are shown for each of the 11 resequenced marmot individuals. We measured number of long runs of homozygosity (> 2MB) as well as the proportion of the genome they covered.

Model	k	LogL	AIC	Model description	Model comparison
SI	4	-9,234.07	18,474.15	Strict isolation	
IM	6	-9,236.98	18,483.97	Isolation-with-migration	SI^{NS}
**AM**	**7**	**-6,238.64**	**12,489.29**	Ancient migration	SI***, IM***, SC+++
SC	7	-9,133.46	18,278.92	Secondary contact	SI***, IM***

Table S3. Model comparison of the migration and demography analysis using DADI, Related to Figure 4. Results of fitting four alternative models of divergence for the comparison of La Grande Sassière and Gsies populations using the SNP dataset. Models are ranked by model category. SI, strict isolation; IM, isolation-with-migration; AM, ancient migration and SC, secondary contact.

k is the number of parameters and AIC the Akaike Information Criterion. Model comparisons within and between classes of models are shown. Nested models were compared using likelihood ratio tests, with subscripts indicating significance levels (abbreviated ***P<0.001; **P <0.01; *P<0.05, NS non-significant). Non-nested models were compared using AIC with relative likelihood of each model compared to the best model L(Mi|Mbest)=exp((AICmin-AICi)/2) (abbreviated +++L(Mi|Mbest)<0.001; ++L(Mi|Mbest) <0.01; +L(Mi|Mbest)<0.05, L(Mi|Mbest)> 0.05 : AIC difference shown) [S4]

Supplemental References

S1. Steppan, S.J., Kenagy, G.J., Zawadzki, C., Robles, R., Lyapunova, E.A., and Hoffmann, R.S. (2011). Molecular data resolve placement of the Olympic marmot and estimate dates of trans-Beringian interchange. J. Mammal. *92*, 1028–1037.

S2. Chao, Q.J., Li, Y.D., Geng, X.X., Zhang, L., Dai, X., Zhang, X., Li, J., and Zhang, H.J. (2014). Complete mitochondrial genome sequence of Marmota himalayana (Rodentia: Sciuridae) and phylogenetic analysis within Rodentia. Genet. Mol. Res. *13*, 2739–2751.

S3. Manichaikul, A., Mychaleckyj, J.C., Rich, S.S., Daly, K., Sale, M., and Chen, W.-M. (2010). Robust relationship inference in genome-wide association studies. Bioinformatics *26*, 2867–2873.

S4. Christe, C., Stölting, K.N., Paris, M., Fraïsse, C., Bierne, N., and Lexer, C. (2017). Adaptive evolution and segregating load contribute to the genomic landscape of divergence in two tree species connected by episodic gene flow. Mol. Ecol. *26*, 59–76.
