# Supplementary material for: Ice-Age Climate Adaptations Trap the Alpine Marmot in a State of Low Genetic Diversity
Source: Curr Biol. 2019 May 20;29(10):1712–1720.e7. doi: 10.1016/j.cub.2019.04.020 (PMC6538971; doi:10.1016/j.cub.2019.04.020)
Supplement: Document S2. Article plus Supplemental Information [file mmc3.pdf]

Report

Current Biology

Ice-Age Climate Adaptations Trap the Alpine Marmot in a State of Low Genetic Diversity

Highlights

- The Alpine marmot is among the least genomically diverse animal species
- Its diversity was lost during consecutive ice-age climate-related events
- An extreme lifestyle hampered the subsequent recovery of genetic variation
- Alpine marmots show why large populations can coexist with very low genetic variation

Authors

Toni I. Gossmann, Achchuthan Shanmugasundram, Stefan Börno, ..., John J. Welch, Bernd Timmermann, Markus Ralser

Correspondence

markus.ralser@crick.ac.uk

In Brief

Despite being highly abundant and well adapted, Gossmann et al. report that the Alpine marmot is among the least genetically diverse animal species. The low diversity is found to be the consequence of consecutive, climate-related events, including long-term extreme niche adaptation, that also greatly retarded the recovery of its genetic diversity.

Gossmann et al., 2019, Current Biology *29*, 1712–1720
May 20, 2019 © 2019 The Author(s). Published by Elsevier Ltd.
https://doi.org/10.1016/j.cub.2019.04.020

Current Biology
Report

Ice-Age Climate Adaptations Trap the Alpine Marmot in a State of Low Genetic Diversity

Toni I. Gossmann,[1,2] Achchuthan Shanmugasundram,[3,4] Stefan Börno,[5] Ludovic Duvaux,[6,7] Christophe Lemaire,[6] Heiner Kuhl,[5,8] Sven Klages,[5] Lee D. Roberts,[9,10] Sophia Schade,[5] Johanna M. Gostner,[11] Falk Hildebrand,[12,13,14] Jakob Vowinckel,[9] Coraline Bichet,[15] Michael Mülleder,[9,16] Enrica Calvani,[3,9] Aleksej Zelezniak,[3,17,18] Julian L. Griffin,[9] Peer Bork,[12,19,20] Dominique Allaine,[21] Aurélie Cohas,[21] John J. Welch,[22,23] Bernd Timmermann,[5,23] and Markus Ralser[3,9,16,23,24,*]
[1]University of Sheffield, Department of Animal and Plant Sciences, Sheffield S10 2TN, UK
[2]Bielefeld University, Department of Animal Behaviour, 33501 Bielefeld, Germany
[3]Molecular Biology of Metabolism Laboratory, The Francis Crick Institute, 1 Midland Road, London NW1 1AT, UK
[4]Centre for Genomic Research, Institute of Integrative Biology, University of Liverpool, Biosciences Building, Crown Street, Liverpool L69 7ZB, UK
[5]Max Planck Institute for Molecular Genetics, Sequencing Core Facility, Ihnestrasse 73, 14195 Berlin, Germany
[6]IRHS, Université d'Angers, INRA, Agrocampus-Ouest, SFR 4207 QuaSaV, 49071 Beaucouzé, France
[7]BIOGECO, INRA, Université de Bordeaux, 69 Route d'Arcachon, 33612 Cestas, France
[8]Department of Ecophysiology and Aquaculture, Leibniz-Institute of Freshwater Ecology and Inland Fisheries, 12587 Berlin, Germany
[9]Department of Biochemistry and Cambridge Systems Biology Centre, University of Cambridge, 80 Tennis Court Road, Cambridge CB2 1GA, UK
[10]Leeds Institute of Cardiovascular and Metabolic Medicine, University of Leeds, Leeds LS2 9JT, UK
[11]Division of Medical Biochemistry, Medical University of Innsbruck, 6020 Innsbruck, Austria
[12]European Molecular Biology Laboratory (EMBL), 69117 Heidelberg, Germany
[13]Earlham Institute, Norwich Research Park, Norwich NR4 7UZ, UK
[14]Gut Health and Microbes Programme, Quadram Institute, Norwich Research Park, Norwich NR4 7UQ, UK
[15]Institute of Avian Research, 26386 Wilhelmshaven, Germany
[16]Department of Biochemistry, Charitè, Am Chariteplatz 1, 10117 Berlin, Germany
[17]Department of Biology and Biological Engineering, Chalmers University of Technology, 412 96 Göteborg, Sweden
[18]Science for Life Laboratory, KTH - Royal Institute of Technology, Stockholm 171 65, Sweden
[19]Max-Delbrück-Centre for Molecular Medicine, 13092 Berlin, Germany
[20]Molecular Medicine Partnership Unit, 69120 Heidelberg, Germany
[21]Université de Lyon, F-69000, Lyon; Université Lyon 1; CNRS, UMR 5558, Laboratoire de Biométrie et Biologie Evolutive, 69622 Villeurbanne, France
[22]Department of Genetics, University of Cambridge, Cambridge CB2 3EH, UK
[23]These authors contributed equally
[24]Lead Contact
*Correspondence: markus.ralser@crick.ac.uk
https://doi.org/10.1016/j.cub.2019.04.020

SUMMARY

Some species responded successfully to prehistoric changes in climate [1, 2], while others failed to adapt and became extinct [3]. The factors that determine successful climate adaptation remain poorly understood. We constructed a reference genome and studied physiological adaptations in the Alpine marmot (*Marmota marmota*), a large ground-dwelling squirrel exquisitely adapted to the "ice-age" climate of the Pleistocene steppe [4, 5]. Since the disappearance of this habitat, the rodent persists in large numbers in the high-altitude Alpine meadow [6, 7]. Genome and metabolome showed evidence of adaptation consistent with cold climate, affecting white adipose tissue. Conversely, however, we found that the Alpine marmot has levels of genetic variation that are among the lowest for mammals, such that deleterious mutations are less effectively purged. Our data rule out typical explanations for low diversity, such as high levels of consanguineous mating, or a very recent bottleneck. Instead, ancient demographic reconstruction revealed that genetic diversity was lost during the climate shifts of the Pleistocene and has not recovered, despite the current high population size. We attribute this slow recovery to the marmot's adaptive life history. The case of the Alpine marmot reveals a complicated relationship between climatic changes, genetic diversity, and conservation status. It shows that species of extremely low genetic diversity can be very successful and persist over thousands of years, but also that climate-adapted life history can trap a species in a persistent state of low genetic diversity.

RESULTS AND DISCUSSION

We sequenced, assembled, and annotated a reference genome for the Alpine marmot (Figure 1A) on the basis of a wild-living male selected from a typical, central Alpine habitat (Mauls

 © 2019 The Author(s). Published by Elsevier Ltd.
This is an open access article under the CC BY license (http://creativecommons.org/licenses/by/4.0/).

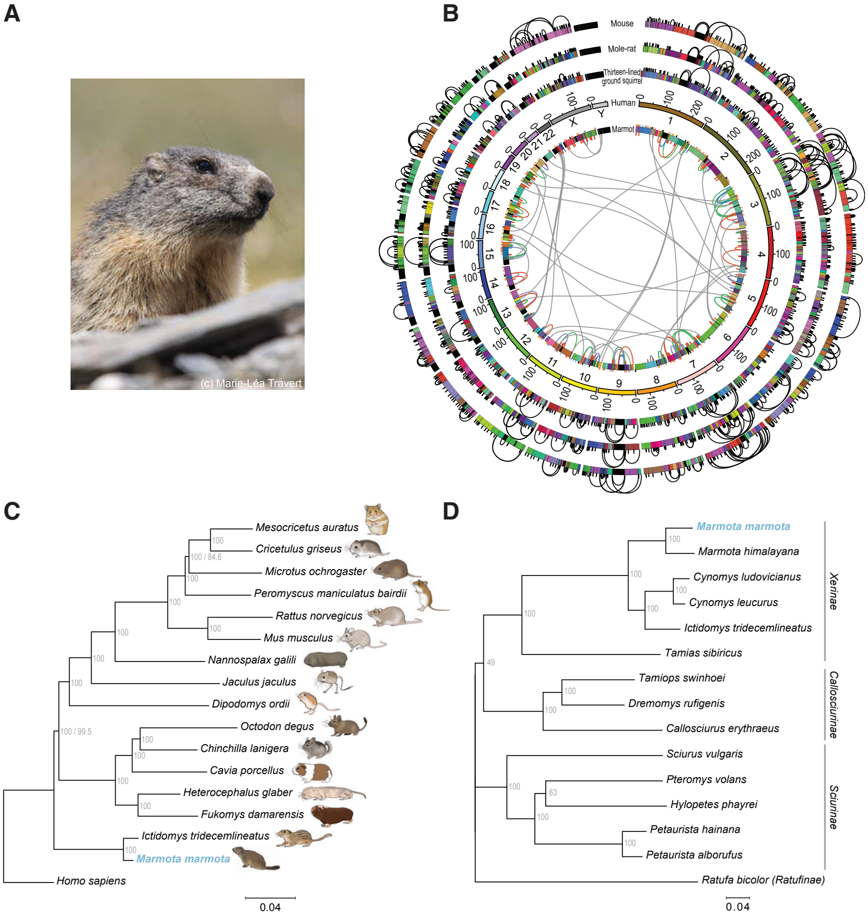

Figure 1. A Slow Rate of Genomic Evolution and the Phylogenetic Relationship of the Alpine Marmot as Revealed by Its Nuclear and Mitochondrial Reference Genome

(A) *Marmota marmota* is a large, ground-dwelling, highly social rodent that has colonized high-altitude meadows across the Alps since the end of the last glaciation in the Quaternary.

(B) Collinearity of the genome assembly generated for *M. marmota marmota* with its close relatives, and that of other rodents. The *M. marmota* genome aligns to a higher fraction of the human genome (outgroup) than to its fellow rodents (i.e., mouse and mole), one of several indicators of a slower rate of genomic evolution. Here, collinear blocks in the human chromosomes are colored at random; small blocks with many colors depict lower N_{50} scaffold length of genome assemblies. Connections indicate collinearity breaks and block rearrangements compared to the human genome (intra-chromosomal only for the plotted rodents, except for *M. marmota* where interchromosomal rearrangements are plotted inside the graph). Colors indicate connections observed in *M. marmota* that are conserved across the sampled rodents (green; n = 72), all rodents except *M. musculus* (blue, n = 13), between *I. tridecemlineatus* and *M. marmota* (purple; n = 57), or that are specific to *M. marmota* (orange; n = 148).

(C) Reconstruction of the phylogenetic tree of Rodentia. The tree is derived from multiple whole-genome alignments of protein-coding and non-coding sequences from available rodent genomes (about 94 Mbp alignment per species). Humans are included as an outgroup. The short branch length of the Alpine marmot since the split from the last common ancestor (LCA) of primates and rodents agrees with the higher fraction of alignable genomic sequence between the Alpine marmot and human compared to Alpine marmot and mouse or mole-rat (Data S1). The scale denotes nucleotide substitutions per site.

(D) A phylogenetic tree for family Sciuridae based on their mitochondrial genomes. The scale denotes nucleotide substitutions per site.

See also Data S1 and Figures S1 and S2.

region, North Italy; Data S1; STAR Methods). Phylogenomic and phylogenetic analyses confirmed the Alpine marmot's relationships to other mammals, rodents, squirrels, and marmots, including the groundhog (*Marmota monax*) [8, 9] (Figures 1B–1D; Data S1; Figure S1). We also identified an unusually large integration of mitochondrial genome into the nuclear genome (nuclear mitochondrial DNA segment, NUMT [10]), which comprises 91% of the mitochondrial genome. The NUMT is well conserved (with 84% similarity to the mtDNA), despite no evidence of functional constraint (no expression on the mRNA level, and many premature stop codons). The nuclear insertion occurred before the common ancestor of *Marmota*, *Ictidomys*, and *Cynomys* (Figures 1D and S2), during which time substitutions occurred at most synonymous sites in the mitochondrial genomes (mitochondrial *Ks* estimates: *Ictidomys-Marmota* 0.48; *Tamias-Marmota* 1.11). This is suggestive of a low rate of nuclear genome evolution, and this was confirmed by a comparison of genome-wide rates in other rodents (median synonymous substitutions per codon/year: *Marmota*:0.0017 versus *Ictidomys*:0.0020, Wilcoxon test $p = 1.6 \times 10^{-20}$; *Marmota/Ictidomys*:0.0029 versus *Mus/Rattus*:0.0042, $p = 6.1 \times 10^{-106}$) and by a collinearity analysis (Figure 1B). The Alpine marmot is therefore characterized by an overall low rate of genomic evolution.

We next searched for genes undergoing exceptional rates of protein evolution specific to hibernating rodents (Figure S3A). Among a group of 1,571 differentially evolving genes, there was specific enrichment for genes related to photoreception (Data S1), and for the metabolic pathway of glycerolipid metabolism, which is essential for the synthesis of triacylglycerols (TAGs), the precursor of fatty acids (Figure 2A). Furthermore, genes in the pathway of fat digestion and absorption, which is essential for the utilization of stored fats, have undergone diversifying rates of evolution within the marmot lineage after the split with the thirteen-lined ground squirrel (*Ictidomys tridecemlineatus*; Figures 2B and S3B).

While the specific enrichment for photoreception was unexpected, the adaptation of the lipidome is plausibly associated with cold temperature adaptation. For physical reasons, a higher degree of unsaturation increases membrane fluidity at low temperature. This adaptation is particularly evident in the white adipose tissue (WAT) that serves hibernating animals for energy

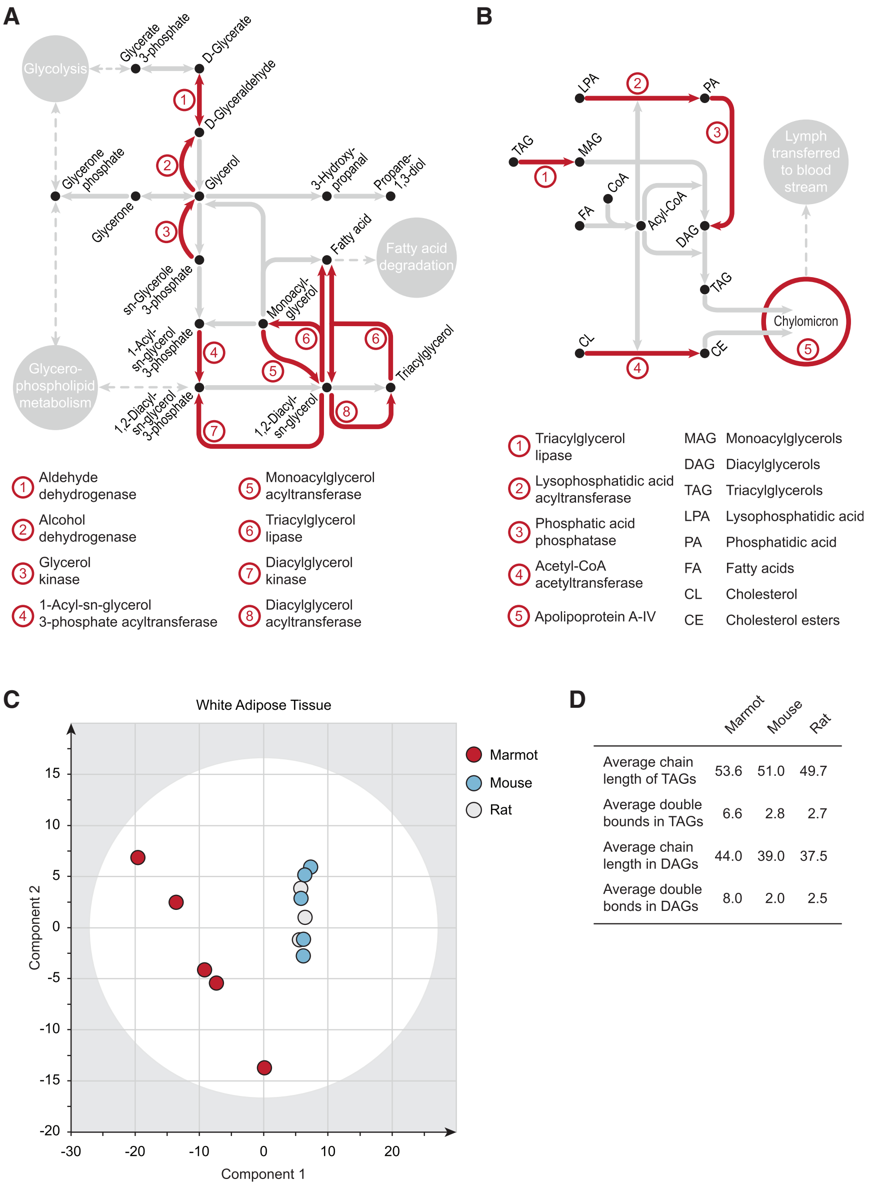

	Marmot	Mouse	Rat
Average chain length of TAGs	53.6	51.0	49.7
Average double bounds in TAGs	6.6	2.8	2.7
Average chain length in DAGs	44.0	39.0	37.5
Average double bonds in DAGs	8.0	2.0	2.5

Figure 2. Genomic Signatures of Metabolic Evolution in the Alpine Marmot, Plausibly Associated with Cold-Climate Adaptation, Are Reflected in an Altered White Adipose Tissue Lipidome

(A) Phylogenetic analysis by maximum likelihood (PAML) followed by tests for functional enrichment identifies biological processes that underwent diversifying evolution in the Alpine marmot lineage. The group of differentially evolving genes in the hibernating rodents (Alpine marmot and thirteen-lined ground squirrel) show significant enrichment at the pathway level for diacylglyceride (DAG) and triacylglyceride (TAG) biosynthetic metabolism. Enzymes under differential selection pressure are highlighted in red.

(B) The Alpine marmot lineage shows specific and significant enrichment of potentially adaptive substitutions in genes required for fatty acid storage, when compared to the thirteen-lined ground squirrel. Enzymes encoding differentially evolving genes are highlighted in red.

(C) Partial least-squares-discriminatory analysis (PLS-DA) of the white adipose tissue (WAT) lipid composition as determined by liquid chromatography-tandem mass spectrometry, comparing mouse, rat, and Alpine marmot WAT. The Alpine marmot WAT is clearly distinguishable from that of rat and mouse

(D) Higher degree of unsaturation, and longer chain lengths in Alpine marmot WAT DAGs and TAGs, as determined by liquid chromatography-tandem mass spectrometry.

See also Data S1, Table S1, and Figure S3.

storage [11], where the levels of polyunsaturated fatty acids are positively correlated with survival during winter hibernation [11–13]. We therefore used mass spectrometry and recorded a lipidome of WAT obtained from Alpine marmots and compared it to that of two non-hibernating rodents: rats (Wistar line) and mice (C57Bl6 line). WAT (Figure 2C; Table S1) TAG and diacylglycerol (DAG) lipids were highly discriminatory, and in the Alpine marmot characterized by greater acyl chain length and unsaturation. Indeed, some changes were substantial: detected up to 4-fold higher level of unsaturation in TAGs and DAGs, the main energy storage lipids that need to be accessed at low temperature (Figure 2D).

A further known adaptation of the Alpine marmot is complete parasite clearance prior to hibernation [14]. While we found no enrichment at the pathway level, four genes involved in anti-parasite defense exhibit significantly elevated molecular substitution rates in comparison to the thirteen-lined ground squirrel. The fastest gene was Interleukin 4 (*dN/dS* of 2.3072, top 1% in a phylogenetic analysis by maximum likelihood [PAML] analysis) (Data S1). The cytokine-cytokine receptor pathway may therefore have undergone adaptive evolution, suggesting that parasite clearance before hibernation might be more than a passive process caused by starvation of the parasites.

We next studied the genome-level diversity of the Alpine marmot. Unexpectedly, the within-individual diversity was found to be remarkably low, with a heterozygosity of 0.12 per kilobase (Figure 3A). To place this result in context, we performed the same analysis on a panel of other mammalian genomes. As well as humans, and close relatives of the marmot, we chose species known for very low heterozygosity, often associated with conservation risk, habitat loss, extreme isolation, or artificial inbreeding [15] (Data S1). Although it is not considered a conservation concern, and despite its high abundance and large geographic range, the Alpine marmot is the least heterozygous among the panel of wild-living animals, including the extreme case of low diversity for a wild-living animal, the Iberian Lynx (Figure 3A). Lower heterozygosity was found only in the lab mouse (129P2/OlaHsd), artificially backcrossed for decades (0.05/kb; Data S1). The Alpine marmot also remains extreme among a large number of species for which heterozygosity values are available in the literature [15].

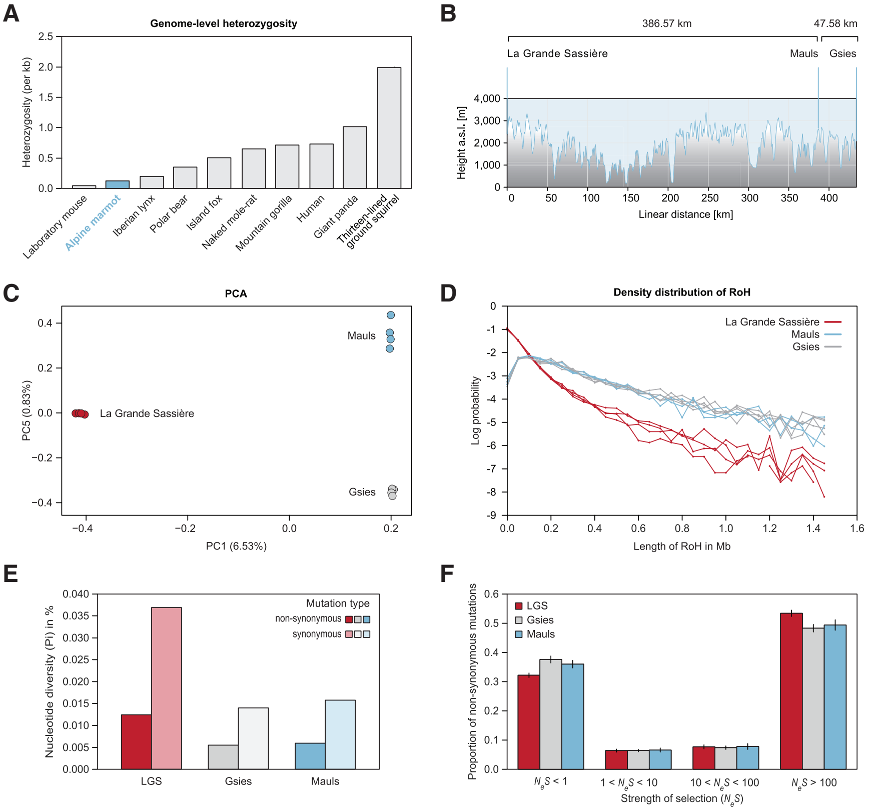

Figure 3. Extremely Low Levels of Genetic Diversity and Impaired Purifying Selection Characterize Different Alpine Marmot Populations

(A) The Alpine marmot genome is characterized by remarkably low heterozygosity at the genome level. The heterozygosity for a panel of other mammalian genomes has been determined by re-mapping the original sequence reads used to assemble their reference genomes, using identical software parameters (Data S1), so that heterozygosity values are directly comparable.

(B) The locations and height profiles of our sampled Alpine marmot populations: Mauls (Italy), Gsies (Italy), and La Grande Sassière (LGS, France).

(C) Principal-component analysis (PCA) of whole-genome genetic diversity (SNPs, including singletons) of animals from Mauls, Gsies, and LGS. PC1 distinguishes the Mauls and Gsies populations from the LGS marmots, while only PC5 separates all three populations. Axes 2–4 mainly describe genetic diversity within the LGS population, which has comparable diversity to the combined sample.

(D) Logarithmic density distributions of runs of homozygosity (RoH) for individuals of the three populations. Distributions are very similar for the Mauls and Gsies populations but different for LGS, and this is well explained by their differences in local breeding sizes. There is little evidence of consanguineous mating, nor of a recent bottleneck recovery.

(E) Differences in the coding diversity (synonymous and nonsynonymous sites) among the three marmot populations. LGS individuals are around three times more diverse that the inner Alpine populations Mauls and Gsies (right).

(F) Distribution of fitness effects of nonsynonymous mutations suggests that more than 30% of nonsynonymous mutations within the Alpine marmot populations are effectively neutral, with a further 5%–10% in the nearly neutral range. There is little variation of fitness effects across populations. Error bars indicate the SE.

See also Data S1, Table S2, and Figures S3 and S4.

An individual may have low levels of genetic diversity for three different reasons: either there is low diversity in their species as a whole or in their local breeding population, or they might have resulted from close inbreeding (i.e., consanguineous mating) within an otherwise diverse population [16]. The latter two explanations were strong possibilities in the Alpine marmot, where breeding takes place in extended family groups, and inbreeding depression has been observed [17–20]. To distinguish between these possibilities, we resequenced a further 11 Alpine marmot individuals (Data S1), both from the reference population, and two additional populations, from Gsies, a neighboring valley less than 50 km East of Mauls and La Grande Sassière (LGS) Nature Reserve, French Alps ~390 km west (Figure 3B), to obtain two male and two female genomes per population. For each population, we calculated the overall levels of diversity at synonymous sites, π_s, across the genome, and typical levels of relatedness (Figures 3E, S4A, and S4B), while, for each individual, we calculated the genome-wide heterozygosity (Figure S4C), runs of homozygosity (RoH) [16] (Figure 3D; Table S2), and the coefficient of inbreeding (Figure S4). Results showed that the three populations were genetically separable (Figure 3C) and suggested clear differences in their effective population sizes. Most notably, the LGS population had over twice the overall genetic diversity (π_S: LGS 0.037%; Mauls 0.016%; Gsies 0.014%; Figures 3E and S4C), and—consistent with this—LGS individuals had higher intra-individual diversity (Figures S4 and 3D). In particular, the Mauls and Gsies marmots had heterozygosity of ~0.1–0.13/kb, similar to the reference animal (Figure 3A), while estimates from LGS marmots were over twice as high (0.29–0.34/kb; Figure S4C), although these values are still extremely low compared to other mammals (Figure 3A).

The data suggest that the smaller local populations (Gsies and Mauls) contain a high proportion of close relatives (Figure S4A), but there was no evidence of consanguineous mating, whose signature is high variance in the total length of homozygous blocks [16] (Figure 3D), and consistently high inbreeding coefficients. Indeed, estimated inbreeding coefficients are skewed toward negative values (Figure S4B). Furthermore, there is evidence that the diversity of the Gsies and Mauls marmots nests within that of the LGS marmots, as would be the case if these populations had "budded" from the larger LGS population [21, 22]. For example, diversity is slightly higher for the LGS sample alone, than for the complete pooled sample (π_S = 0.037% versus π_S = 0.033%), and for the mitochondrial genomes, all Gsies and Mauls marmots descend from the most recent common ancestor of the LGS sample (Figure S3C). This scenario is also consistent with the Gsies and Mauls marmots being separable only by the fifth principal component (Figure 3C). Taken together, then, the low levels of diversity within individual marmots are partly due to population structure but also reflect a low effective population size in the species as a whole.

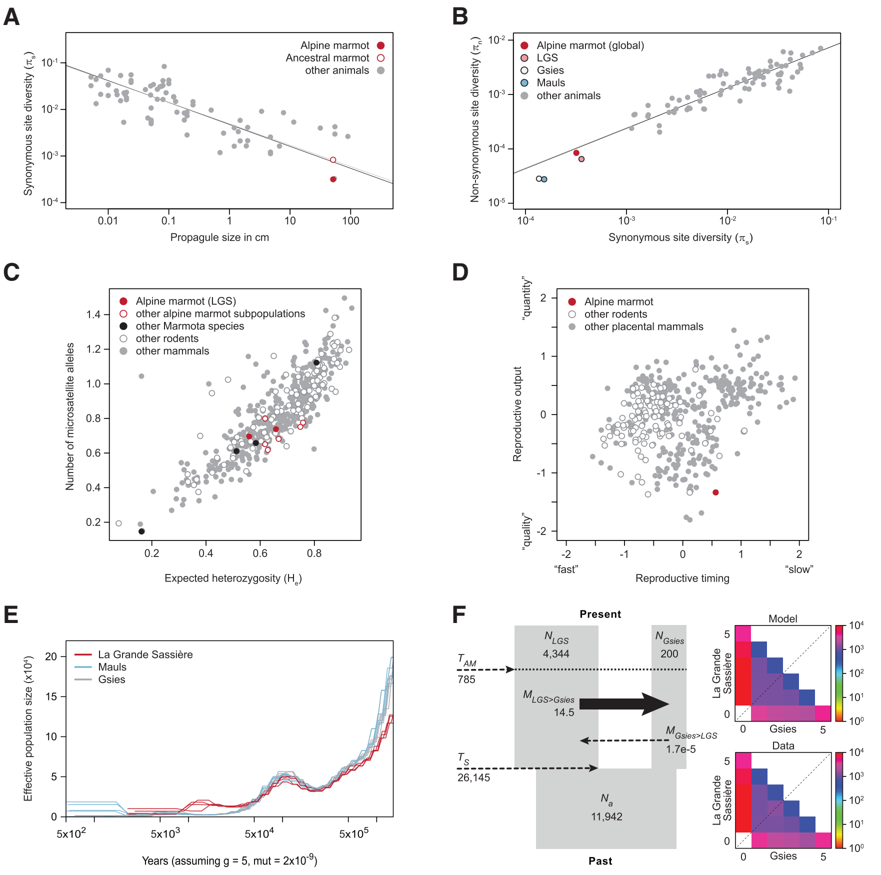

Figure 4. The Low Genomic Diversity in the Alpine Marmot Is Explained by Its Life History and a Lack of Recovery from a Bottleneck that Coincides with the Climate Shifts of the Pleistocene

(A) The genetic diversity of the Alpine marmot is predictable from its life history. Species-wide synonymous site diversity from a wide range of animal species [24] is plotted against their "propagule size" (i.e., the size in centimeters of the dispersing life stage). The delayed dispersal of the Alpine marmot, and its large adult body size, yields a very large propagule size [25], consistent with the observed low diversity (filled red point). The diversity inferred for the ancestral marmot population at the end of the Pleistocene (empty red point; F) fits this prediction even more closely. The correlations observed are very similar whether the marmot data are excluded (gray lines) or included (black lines).

(B) The strength of purifying selection on amino acid variation in Alpine marmots corresponds to their low effective population size revealed from a pattern consistent across diverse animal species. Data from the Alpine marmot (colored points) have been added to the data of [24]. The correlations are very similar whether the marmot data are excluded (gray lines) or included (black lines).

(C) Microsatellite diversity in different Alpine marmot populations compared to many other species of mammals, including other marmot and rodent species. The number of microsatellite alleles (y axis) is plotted against the expected heterozygosity (x axis). Populations of the Alpine marmot from LGS are shown as red points, and estimates from other subpopulations of the same species, also from the French alps, are shown in as empty red bordered circles. Other species in the genus *Marmota* are shown as black filled circles, including the threatened *M. vancouverensis*, which appears at the bottom left of the graph. In difference to the genome-wide diversity, for which the Marmot is an extreme (A and B), it has a typical diversity in these sites that are characterized by a higher spontaneous mutation rate.

(D) Life history of the Alpine marmot (red point) in comparison to other Eutherian mammals (data from [26]). After correcting for body mass, much of the variance in mammalian life histories can be captured by two factors [27]: "reproductive output" (in which species vary according to their investment in offspring "quality" versus "quantity") and "reproductive timing" (in which species vary on a "fast-slow" continuum). The Alpine marmot appears as an extreme outlier.

(E) Pairwise sequential Markovian coalescent (PSMC) analysis reveals details of the genetic past of the Alpine marmot. Evident is a decline in the LGS population after the last glacial maximum, consistent with independent evidence from the fossil record. Earlier events might suggest a longer-term decline but are more likely attributable to partial isolation between breeding populations (in which case earlier rates of coalescence reflect rates of gene flow and not $N_e s$).

(F) The ancient migration (*AM*) is the most likely demographic scenario for the Gsies and La Grande Sassière (LGS) populations inferred from the joint site frequency spectrum (SFS). This model predicts that a large ancestral population split up ~26,145 ya into two smaller daughter populations. Gene flow between these two populations ceased ~785 ya and was strongly asymmetrical with most migrants going from the large LGS population to the very small Gsies population. The joint SFS for the LGS and Gsies populations was obtained from 178,098 SNPs (bottom right) and shows strong visual agreement with the maximum-likelihood SFS under the model (top right).

See also Table S3.

When the effective population size is low, natural selection can become less effective. This situation was evident in the Alpine marmot genome. First, ratios of amino acid changing to synonymous polymorphism are high (π_N/π_S: LGS 33.7%; Mauls 37.5%; Gsies 39.0%; combined sample: 34.6%; Figure 3E). Second, the distribution of fitness effects [23] suggests that 30%–40% of amino acid variants are under ineffective purifying selection, and a further 5%–10% in the "slightly deleterious" range ($1 < N_e s < 10$), where selection might become ineffective, following any further drop in N_e (Figure 3F).

Given the fact that the Alpine marmot is well adapted and highly abundant, these results initially appeared surprising. To explain the low diversity, we next considered the marmot's unusual life history, which differs, in part substantially, from that of typical (Alpine) mammals. Previous work has shown that species-wide diversity across a broad range of animal species is well predicted by their "propagule size," i.e., the size of the life stage that leaves its parents and disperses [24]. The Alpine marmot fits this pattern remarkably well (Figure 4A). Similarly, the levels of effective selective constraint, π_N/π_S, are very similar to those that would be predicted from previously observed correlations with π_S (Figure 4B). In both cases, the Alpine marmot is an extreme case compared to all other sampled animal species, with the lowest π_S and the highest π_N/π_S, but this reflects the extremity of its life history. Its unusually large propagule size is a result of both its large adult body size and

its delayed, adult dispersal, consistent with its form of proto-cooperative breeding. Even after correcting for body size, the Alpine marmot is extreme among mammals, and especially rodents, in the extent to which it invests in a small number of "high-quality" offspring (Figure 4D). These traits are plausibly adaptations to cold-climate habitation [25, 26].

While correlations of genetic diversity with life history are well established, it remains unclear exactly why they hold. One possibility is that a species' life history has a major influence on its response to demographic perturbations, such as major changes in climate [24]. Such events are historical contingencies, but different species might respond in predictably different ways, with predictable consequences for their genetic variation. The Alpine marmot is a useful case study here, because its fossil record provides clear evidence of a major demographic perturbation, associated with climate change. In particular, the species underwent a large range contraction toward the end of the Pleistocene, after the last glacial maximum [28]. The shift from the steppe to Alpine habitats might also have brought increasing isolation, exacerbated by the expansion of forests that replaced the cold steppe of the Pleistocene, and that are incompatible with the Alpine marmot's lifestyle. To shed light on the demographic history and its effects on genetic variation, we reconstructed the effective population size over time, using the pairwise sequentially Markovian coalescent (PSMC). Toward the end of the Pleistocene (left-hand side of the plot in Figure 4E) the PSMCs confirmed the signature of the known range contraction, with a dip in the LGS population size between the last glacial maximum (~20 ka), and the start of the Holocene (11.65 ka). This signature is messy, but this is as expected in a structured population [29, 30]. To investigate the more recent demographic events, we analyzed the genome-wide site frequency spectrum of the two least connected populations (LGS and Gsies; Figure 4F; Table S3). After comparing several different demographic models, we inferred that these populations descended from a single ancestral population, that was roughly three times larger than the current populations (11,942 versus 4,544 breeding individuals). The population split and decline is dated at 26 kybp, with confidence intervals overlapping the last glacial maximum. We also infer strong and asymmetrical gene flow, continuing long after the split. Our findings are consistent with a post-glaciation colonization hypothesis progressing from the West to East Alps that matches the fossil record [21, 22].

By combining the population size estimates (Figure 4F), and our measure of current diversity, π_S, we can estimate the genetic diversity of this ancestral marmot population (empty red point, Figure 4A). The inferred ancestral diversity is remarkably close to the value that would be predicted from the marmot propagule size (inferred ancestral, $\pi_S = 8.6 \times 10^{-4}$; predicted from propagule size, $\pi_S = 7.7 \times 10^{-4}$).

If the low genetic diversity of the Alpine marmot is due to a slow recovery from past demographic events, then we might predict to see signs of an ongoing recovery in the data. No such evidence was found in the genome-wide data: neither RoH, nor the site frequency spectrum showed signs of recovery from a bottleneck (Figure 3D; Tajima's D at synonymous sites = 0.45). However, in regions of the genome with typical mutation rates, the recovery of diversity might be glacially slow. In this case, a recovery would leave its signature only in rare regions with very high mutation rates, such as microsatellite loci [24, 31]. Indeed, in stark contrast to their low genome-wide diversity, the microsatellite diversity of Alpine marmots was found to be typical, of mammals as a whole, of rodents, and of the genus *Marmota* (Figure 4C). Levels of microsatellite diversity in this genus are conspicuously lower only for *Marmota vancouverensis*, which lives only in the limited habitat of Vancouver island, and is the sole marmot species under threat of extinction [32].

Taken as a whole, these results have two contrasting implications for our understanding of extinction risk. First, it is clear that low levels of genome-wide variation, on their own, need not imply an imminent threat of extinction. The Alpine marmot has persisted successfully, with remarkably low levels of genetic variation, for tens of thousands of years. Conversely, however, there is no cause for complacency. If adaptation to future environmental change does require abundant genomic variation, then populations may be unable to respond, even if they are characterized by high levels of microsatellite diversity and large population size. All species may undergo occasional demographic fluctuations, but factors such as low fecundity, long generation time, and a slow rate of genome evolution would cause some species to take much longer to replenish their genetic diversity after these events. All of these factors are characteristic of the Alpine marmot, very plausibly due to its niche adaptation (Figures 1B, 1C, and 4D), and our data suggest that even their large population size was not sufficient to regenerate diversity over thousands of years. Hence, if low genetic variation is a contributory factor to extinction risk, not only small but also large populations can be at risk, if their life history traps them permanently in a state of low genetic diversity.

STAR★METHODS

Detailed methods are provided in the online version of this paper and include the following:

- KEY RESOURCES TABLE
- CONTACT FOR REAGENT AND RESOURCE SHARING
- EXPERIMENTAL MODEL AND SUBJECT DETAILS
    - Sample collection
- METHOD DETAILS
    - General approach
    - DNA extraction, genomic sequencing and resequencing
    - Assembly of a reference genome for the Alpine marmot
    - Phylogenomic tree for rodent species
    - Spleen RNA extraction and RNASeq
    - Repeat annotation
    - Gene model prediction
    - Functional annotation
    - Non-coding RNA annotation
    - Mitochondrial genome annotation
    - Sciuridae phylogeny based on mtDNA conservation
    - Protein coding sequence alignment across species
    - Inferring positive natural selection on protein coding genes
    - Gene set enrichment analysis
    - Variant impact analysis between Alpine marmot and thirteen-lined ground squirrel

- ○ Heterozygosity analysis across species and SNP calling of Alpine marmot individuals
- ○ Dendrogram-based Alpine marmot population analysis
- ○ Demographic inference with PSMC
- ○ Diffusion Approximation for Demographic Inference (DADI) and PCA
- ○ Coding diversity analysis
- ○ Microsatellite diversity across the mammals
- ○ Life history of the Alpine marmot in comparison to other Eutherian mammals
- ○ Lipidomics
- QUANTIFICATION AND STATISTICAL ANALYSIS
- DATA AND SOFTWARE AVAILABILITY

SUPPLEMENTAL INFORMATION

Supplemental Information can be found online at https://doi.org/10.1016/j.cub.2019.04.020.

ACKNOWLEDGMENTS

We are grateful to Florian Winkler, Heinrich Aukenthaler, Erhard Seehauser, and Gottfried Hopfgartner (Forestry and Hunting Authorities South Tyrol, or Jagdrevier Mauls, Bolzano Province, Italy) for their support in our study of Alpine marmot biology in their wild habitats of Mauls and Gsies (Italy). Further, we thank Dorothee Huchon (Department of Zoology, Tel Aviv University, Israel) for help in rodent phylogenies; Love Dalén (Swedish Museum of Natural History, Sweden), Nicolas Bierne, and Aylwyn Scally (University of Cambridge) for key discussions related to the genomics part of the manuscript; and Mark Wilson (The Francis Crick Institute, UK) for help with parasite defense genes. We are grateful to Kerstin Lindblad-Toh and the Broad Institute (MA, USA) vertebrate genome team for providing the genome sequence of the thirteen-lined ground squirrel. We further thank Jenny Barna (University of Cambridge, UK) for help with software tools, Y. Yuan as well as the European Molecular Biology Laboratory (EMBL) IT core facility and for managing high-performance computing resources, and Bogoljub Trickovic for help with mining the microsatellite database. Further, we thank M.L. Travert (France) for providing photographs of wild-living Alpine marmot in the La Grande Sassière National Park (France) (Figure 1A). This work was supported by the Francis Crick Institute, which receives its core funding from Cancer Research UK (FC001134), the UK Medical Research Council (FC001134), and the Wellcome Trust (FC001134). C.B. and A.C. are supported by the Agence Nationale de la Recherche (project ANR-13-JSV7-0005) and the Centre National de la Recherche Scientifique (CNRS), and C.B. is supported by the Rhône-Alpes region (grant 15.005146.01). L.D. is supported by Agence Nationale de la Recherche (project ANR-12-ADAP-0009). T.I.G. is supported by a Leverhulme Early Career Fellowship (grant ECF-2015-453) and an NERC grant (NE/N013832/1). J.M.G. is supported by a Hertha Firnberg Fellowship (FWF T703). L.D.R. is supported by the Diabetes UK RD Lawrence Fellowship (16/0005382).

AUTHOR CONTRIBUTIONS

M.R., B.T., J.J.W., and T.I.G. designed and supervised the study; M.R., J.J.W., and T.I.G. wrote the paper with contributions from all authors; J.J.W., T.I.G., S.S., and B.T. carried out data presentation; M.R., J.M.G., J.V., C.B., D.A., A.C., E.C., and M.M. conducted marmot observations, sample collection, and sample processing; B.T. led genome sequencing and assembly with contributions from H.K., A.S., S.K., S.B., and B.T.; A.S. and H.K. led genome annotation and data handling with contributions from J.J.W., A.S., H.K., S.K., S.B., F.H., P.B., and B.T.; H.K. and J.J.W. constructed phylogenies; A.S., T.I.G., J.J.W., and L.D. conducted analysis of genetic variability; T.I.G. and J.J.W. conducted PSMC modeling; C.L. and L.D. conducted diffusion-based demographic inferences; J.J.W., A.S., T.I.G., and H.K. conducted gene evolution analysis; and L.D.R. and J.L.G. conducted lipidomics.

DECLARATION OF INTERESTS

The authors declare no competing interests.

Received: December 6, 2018
Revised: February 16, 2019
Accepted: April 9, 2019
Published: May 9, 2019

REFERENCES

1. Robson, K.M., Lamb, C.T., and Russello, M.A. (2015). Low genetic diversity, restricted dispersal, and elevation-specific patterns of population decline in American pikas in an atypical environment. J. Mammal. *97*, 464–472.
2. Kumar, V., Kutschera, V.E., Nilsson, M.A., and Janke, A. (2015). Genetic signatures of adaptation revealed from transcriptome sequencing of Arctic and red foxes. BMC Genomics *16*, 585.
3. Nogués-Bravo, D., Rodríguez, J., Hortal, J., Batra, P., and Araújo, M.B. (2008). Climate change, humans, and the extinction of the woolly mammoth. PLoS Biol. *6*, e79.
4. Bichet, C., Allainé, D., Sauzet, S., and Cohas, A. (2016). Faithful or not: direct and indirect effects of climate on extra-pair paternities in a population of Alpine marmots. Proc. Biol. Sci. *283*, 20162240.
5. Tafani, M., Cohas, A., Bonenfant, C., Gaillard, J.-M., and Allainé, D. (2013). Decreasing litter size of marmots over time: a life history response to climate change? Ecology *94*, 580–586.
6. Couturier, M. (1955). Acclimatation et acclimatement de la Marmotte des Alpes, Marmota marmota (Linné 1758), dans les Pyrénées françaises. Saugetierkdl. Mitt. *3*, 105–107.
7. Besson, J.P. (1971). Introduction de la marmotte dans les Pyrénées occidentales. CR du 96ème Congrès des Sociétés Savantes, Toulouse *3*, 397–399.
8. Fabre, P.H., Hautier, L., Dimitrov, D., and Douzery, E.J.P. (2012). A glimpse on the pattern of rodent diversification: a phylogenetic approach. BMC Evol. Biol. *12*, 88.
9. Blanga-Kanfi, S., Miranda, H., Penn, O., Pupko, T., DeBry, R.W., and Huchon, D. (2009). Rodent phylogeny revised: analysis of six nuclear genes from all major rodent clades. BMC Evol. Biol. *9*, 71.
10. Hazkani-Covo, E., Zeller, R.M., and Martin, W. (2010). Molecular poltergeists: mitochondrial DNA copies (numts) in sequenced nuclear genomes. PLoS Genet. *6*, e1000834.
11. Cochet, N., Georges, B., Meister, R., Florant, G.L., and Barré, H. (1999). White adipose tissue fatty acids of Alpine marmots during their yearly cycle. Lipids *34*, 275–281.
12. Frank, C.L. (1992). The influence of dietary fatty acids on hibernation by golden-mantled ground squirrels (Spermophilus lateralis). Physiol. Zool *65*, 906–920.
13. Bruns, U., Frey-Roos, F., Pudritz, S., Tataruch, F., Ruf, T., and Arnold, W. (2000). Essential fatty acids: their impact on free-living Alpine marmots (Marmota marmota). In Life in the Cold, P.D.G. Heldmaier, and M. Klingenspor, eds. (Springer Berlin Heidelberg), pp. 215–222.
14. Bohr, M., Brooks, A.R., and Kurtz, C.C. (2014). Hibernation induces immune changes in the lung of 13-lined ground squirrels (Ictidomys tridecemlineatus). Dev. Comp. Immunol. *47*, 178–184.
15. Robinson, J.A., Ortega-Del Vecchyo, D., Fan, Z., Kim, B.Y., vonHoldt, B.M., Marsden, C.D., Lohmueller, K.E., and Wayne, R.K. (2016). Genomic flatlining in the endangered island fox. Curr. Biol. *26*, 1183–1189.
16. Ceballos, F.C., Joshi, P.K., Clark, D.W., Ramsay, M., and Wilson, J.F. (2018). Runs of homozygosity: windows into population history and trait architecture. Nat. Rev. Genet. *19*, 220–234.
17. Arnold, W. (1990). The evolution of marmot sociality: I. Why disperse late? Behav. Ecol. Sociobiol. *27*, 229–237.

18. Goossens, B., Chikhi, L., Taberlet, P., Waits, L.P., and Allainé, D. (2001). Microsatellite analysis of genetic variation among and within Alpine marmot populations in the French Alps. Mol. Ecol. *10*, 41–52.
19. Cohas, A., Yoccoz, N.G., Da Silva, A., Goossens, B., and Allainé, D. (2005). Extra-pair paternity in the monogamous alpine marmot (Marmota marmota): the roles of social setting and female mate choice. Behav. Ecol. Sociobiol. *59*, 597–605.
20. Nichols, H.J. (2017). The causes and consequences of inbreeding avoidance and tolerance in cooperatively breeding vertebrates. J. Zool. (Lond.) *303*, 1–14.
21. Preleuthner, M., Pinsker, W., Kruckenhauser, L., Miller, W.J., and Prosl, H. (1995). Alpine marmots in Austria. The present population structure as a result of the postglacial distribution history. Acta Theriol. (Warsz.) *40*, 87–100.
22. Kruckenhauser, L., and Pinsker, W. (2004). Microsatellite variation in autochthonous and introduced populations of the Alpine marmot (Marmota marmota) along a European west–east transect. J. Zoological Syst. Evol. Res. *42*, 19–26.
23. Keightley, P.D., and Eyre-Walker, A. (2007). Joint inference of the distribution of fitness effects of deleterious mutations and population demography based on nucleotide polymorphism frequencies. Genetics *177*, 2251–2261.
24. Romiguier, J., Gayral, P., Ballenghien, M., Bernard, A., Cahais, V., Chenuil, A., Chiari, Y., Dernat, R., Duret, L., Faivre, N., et al. (2014). Comparative population genomics in animals uncovers the determinants of genetic diversity. Nature *515*, 261–263.
25. Ge, D.Y., Liu, X., Lv, X.F., Zhang, Z.Q., Xia, L., and Yang, Q.S. (2013). Historical biogeography and body form evolution of ground squirrels (Sciuridae: Xerinae). Evol. Biol. *41*, 99–114.
26. Jones, K.E., Bielby, J., Cardillo, M., Fritz, S.A., O'Dell, J., Orme, C.D.L., Safi, K., Sechrest, W., Boakes, E.H., Carbone, C., et al. (2009). PanTHERIA: a species-level database of life history, ecology, and geography of extant and recently extinct mammals. Ecology *90*, 2648–2648.
27. Bielby, J., Mace, G.M., Bininda-Emonds, O.R.P., Cardillo, M., Gittleman, J.L., Jones, K.E., Orme, C.D.L., and Purvis, A. (2007). The fast-slow continuum in mammalian life history: an empirical reevaluation. Am. Nat. *169*, 748–757.
28. Zimina, R.P., and Gerasimov, I.P. (1973). The periglacial expansion of marmots (Marmota) in Middle Europe during Late Pleistocene. J. Mammal. *54*, 327–340.
29. Chikhi, L., Rodríguez, W., Grusea, S., Santos, P., Boitard, S., and Mazet, O. (2018). The IICR (inverse instantaneous coalescence rate) as a summary of genomic diversity: insights into demographic inference and model choice. Heredity *120*, 13–24.
30. Li, H., and Durbin, R. (2011). Inference of human population history from individual whole-genome sequences. Nature *475*, 493–496.
31. Pannell, J.R., and Charlesworth, B. (2000). Effects of metapopulation processes on measures of genetic diversity. Philos. Trans. R. Soc. Lond. B Biol. Sci. *355*, 1851–1864.
32. Roach, N. (2017). Marmota vancouverensis. (IUCN Red List of Threatened Species). https://doi.org/10.2305/iucn.uk.2017-2.rlts.t12828a22259184.
33. Ralser, M., Kuhl, H., Ralser, M., Werber, M., Lehrach, H., Breitenbach, M., and Timmermann, B. (2012). The Saccharomyces cerevisiae W303-K6001 cross-platform genome sequence: insights into ancestry and physiology of a laboratory mutt. Open Biol. *2*, 120093.
34. Cantarel, B.L., Korf, I., Robb, S.M.C., Parra, G., Ross, E., Moore, B., Holt, C., Sánchez Alvarado, A., and Yandell, M. (2008). MAKER: an easy-to-use annotation pipeline designed for emerging model organism genomes. Genome Res. *18*, 188–196.
35. Leggett, R.M., Clavijo, B.J., Clissold, L., Clark, M.D., and Caccamo, M. (2014). NextClip: an analysis and read preparation tool for Nextera Long Mate Pair libraries. Bioinformatics *30*, 566–568.
36. Mueller, J.C., Kuhl, H., Timmermann, B., and Kempenaers, B. (2016). Characterization of the genome and transcriptome of the blue tit Cyanistes caeruleus: polymorphisms, sex-biased expression and selection signals. Mol. Ecol. Resour. *16*, 549–561.
37. Kiełbasa, S.M., Wan, R., Sato, K., Horton, P., and Frith, M.C. (2011). Adaptive seeds tame genomic sequence comparison. Genome Res. *21*, 487–493.
38. Frankl-Vilches, C., Kuhl, H., Werber, M., Klages, S., Kerick, M., Bakker, A., de Oliveira, E.H., Reusch, C., Capuano, F., Vowinckel, J., et al. (2015). Using the canary genome to decipher the evolution of hormone-sensitive gene regulation in seasonal singing birds. Genome Biol. *16*, 19.
39. Altschul, S.F., Gish, W., Miller, W., Myers, E.W., and Lipman, D.J. (1990). Basic local alignment search tool. J. Mol. Biol. *215*, 403–410.
40. Grabherr, M.G., Russell, P., Meyer, M., Mauceli, E., Alföldi, J., Di Palma, F., and Lindblad-Toh, K. (2010). Genome-wide synteny through highly sensitive sequence alignment: Satsuma. Bioinformatics *26*, 1145–1151.
41. Krzywinski, M., Schein, J., Birol, I., Connors, J., Gascoyne, R., Horsman, D., Jones, S.J., and Marra, M.A. (2009). Circos: an information aesthetic for comparative genomics. Genome Res. *19*, 1639–1645.
42. Blanchette, M., Kent, W.J., Riemer, C., Elnitski, L., Smit, A.F.A., Roskin, K.M., Baertsch, R., Rosenbloom, K., Clawson, H., Green, E.D., et al. (2004). Aligning multiple genomic sequences with the threaded blockset aligner. Genome Res. *14*, 708–715.
43. Price, M.N., Dehal, P.S., and Arkin, A.P. (2010). FastTree 2–approximately maximum-likelihood trees for large alignments. PLoS ONE *5*, e9490.
44. Kim, D., Pertea, G., Trapnell, C., Pimentel, H., Kelley, R., and Salzberg, S.L. (2013). TopHat2: accurate alignment of transcriptomes in the presence of insertions, deletions and gene fusions. Genome Biol. *14*, R36.
45. Trapnell, C., Roberts, A., Goff, L., Pertea, G., Kim, D., Kelley, D.R., Pimentel, H., Salzberg, S.L., Rinn, J.L., and Pachter, L. (2012). Differential gene and transcript expression analysis of RNA-seq experiments with TopHat and Cufflinks. Nat. Protoc. *7*, 562–578.
46. Yates, A., Akanni, W., Amode, M.R., Barrell, D., Billis, K., Carvalho-Silva, D., Cummins, C., Clapham, P., Fitzgerald, S., Gil, L., et al. (2016). Ensembl 2016. Nucleic Acids Res. *44* (D1), D710–D716.
47. UniProt Consortium (2015). UniProt: a hub for protein information. Nucleic Acids Res. *43*, D204–D212.
48. O'Leary, N.A., Wright, M.W., Brister, J.R., Ciufo, S., Haddad, D., McVeigh, R., Rajput, B., Robbertse, B., Smith-White, B., Ako-Adjei, D., et al. (2016). Reference sequence (RefSeq) database at NCBI: current status, taxonomic expansion, and functional annotation. Nucleic Acids Res. *44* (D1), D733–D745.
49. Stanke, M., Diekhans, M., Baertsch, R., and Haussler, D. (2008). Using native and syntenically mapped cDNA alignments to improve de novo gene finding. Bioinformatics *24*, 637–644.
50. Korf, I. (2004). Gene finding in novel genomes. BMC Bioinformatics *5*, 59.
51. Gotoh, O. (2008). A space-efficient and accurate method for mapping and aligning cDNA sequences onto genomic sequence. Nucleic Acids Res. *36*, 2630–2638.
52. Gene Ontology Consortium (2015). Gene Ontology Consortium: going forward. Nucleic Acids Res. *43*, D1049–D1056.
53. Jones, P., Binns, D., Chang, H.-Y., Fraser, M., Li, W., McAnulla, C., McWilliam, H., Maslen, J., Mitchell, A., Nuka, G., et al. (2014). InterProScan 5: genome-scale protein function classification. Bioinformatics *30*, 1236–1240.
54. Kanehisa, M., Sato, Y., Kawashima, M., Furumichi, M., and Tanabe, M. (2016). KEGG as a reference resource for gene and protein annotation. Nucleic Acids Res. *44* (D1), D457–D462.
55. Powell, S., Forslund, K., Szklarczyk, D., Trachana, K., Roth, A., Huerta-Cepas, J., Gabaldón, T., Rattei, T., Creevey, C., Kuhn, M., et al. (2013). eggNOG v4. 0: nested orthology inference across 3686 organisms. Nucleic Acids Res. *42*, D231–D239.
56. Schattner, P., Brooks, A.N., and Lowe, T.M. (2005). The tRNAscan-SE, snoscan and snoGPS web servers for the detection of tRNAs and snoRNAs. Nucleic Acids Res. *33*, W686–W689.

57. Jin, G.-Y., Huang, H.-J., and Zhang, M.-H. (2016). The complete mitochondrial genome of Daurian ground squirrel, Spermophilus dauricus. Mitochondrial DNA A. DNA Mapp. Seq. Anal. *27*, 2848–2849.

58. Edgar, R.C. (2004). MUSCLE: multiple sequence alignment with high accuracy and high throughput. Nucleic Acids Res. *32*, 1792–1797.

59. Stamatakis, A. (2014). RAxML version 8: a tool for phylogenetic analysis and post-analysis of large phylogenies. Bioinformatics *30*, 1312–1313.

60. Mercer, J.M., and Roth, V.L. (2003). The effects of Cenozoic global change on squirrel phylogeny. Science *299*, 1568–1572.

61. Helgen, K.M., Russell Cole, F., Helgen, L.E., and Wilson, D.E. (2009). Generic revision in the Holarctic ground squirrel GenusSpermophilus. J. Mammal. *90*, 270–305.

62. Steppan, S.J., Storz, B.L., and Hoffmann, R.S. (2004). Nuclear DNA phylogeny of the squirrels (Mammalia: Rodentia) and the evolution of arboreality from c-myc and RAG1. Mol. Phylogenet. Evol. *30*, 703–719.

63. Wu, M., Chatterji, S., and Eisen, J.A. (2012). Accounting for alignment uncertainty in phylogenomics. PLoS ONE *7*, e30288.

64. Suyama, M., Torrents, D., and Bork, P. (2006). PAL2NAL: robust conversion of protein sequence alignments into the corresponding codon alignments. Nucleic Acids Res. *34*, W609–W612.

65. Yang, Z. (1997). PAML: a program package for phylogenetic analysis by maximum likelihood. Comput. Appl. Biosci. *13*, 555–556.

66. Benjamini, Y., and Hochberg, Y. (1995). Controlling the false discovery rate: a practical and powerful approach to multiple testing. J. R. Stat. Soc. Series B Stat. Methodol. *57*, 289–300.

67. Wang, J., Duncan, D., Shi, Z., and Zhang, B. (2013). WEB-based GEne SeT AnaLysis Toolkit (WebGestalt): update 2013. Nucleic Acids Res. *41*, W77–W83.

68. Li, H., Handsaker, B., Wysoker, A., Fennell, T., Ruan, J., Homer, N., Marth, G., Abecasis, G., and Durbin, R.; 1000 Genome Project Data Processing Subgroup (2009). The Sequence Alignment/Map format and SAMtools. Bioinformatics *25*, 2078–2079.

69. Cingolani, P., Platts, A., Wang, L., Coon, M., Nguyen, T., Wang, L., Land, S.J., Lu, X., and Ruden, D.M. (2012). A program for annotating and predicting the effects of single nucleotide polymorphisms, SnpEff: SNPs in the genome of Drosophila melanogaster strain w1118; iso-2; iso-3. Fly (Austin) *6*, 80–92.

70. Szklarczyk, D., Franceschini, A., Wyder, S., Forslund, K., Heller, D., Huerta-Cepas, J., Simonovic, M., Roth, A., Santos, A., Tsafou, K.P., et al. (2015). STRING v10: protein-protein interaction networks, integrated over the tree of life. Nucleic Acids Res. *43*, D447–D452.

71. Li, H., and Durbin, R. (2009). Fast and accurate short read alignment with Burrows-Wheeler transform. Bioinformatics *25*, 1754–1760.

72. Langmead, B., and Salzberg, S.L. (2012). Fast gapped-read alignment with Bowtie 2. Nat. Methods *9*, 357–359.

73. McKenna, A., Hanna, M., Banks, E., Sivachenko, A., Cibulskis, K., Kernytsky, A., Garimella, K., Altshuler, D., Gabriel, S., Daly, M., and DePristo, M.A. (2010). The Genome Analysis Toolkit: a MapReduce framework for analyzing next-generation DNA sequencing data. Genome Res. *20*, 1297–1303.

74. Danecek, P., Auton, A., Abecasis, G., Albers, C.A., Banks, E., DePristo, M.A., Handsaker, R.E., Lunter, G., Marth, G.T., Sherry, S.T., et al.; 1000 Genomes Project Analysis Group (2011). The variant call format and VCFtools. Bioinformatics *27*, 2156–2158.

75. Narasimhan, V., Danecek, P., Scally, A., Xue, Y., Tyler-Smith, C., and Durbin, R. (2016). BCFtools/RoH: a hidden Markov model approach for detecting autozygosity from next-generation sequencing data. Bioinformatics *32*, 1749–1751.

76. Quinlan, A.R., and Hall, I.M. (2010). BEDTools: a flexible suite of utilities for comparing genomic features. Bioinformatics *26*, 841–842.

77. Schwartz, O.A., Armitage, K.B., and Van Vuren, D. (1998). A 32-year demography of yellow-bellied marmots (Marmota flaviventris). J. Zool. (Lond.) *246*, 337–346.

78. DePristo, M.A., Banks, E., Poplin, R., Garimella, K.V., Maguire, J.R., Hartl, C., Philippakis, A.A., del Angel, G., Rivas, M.A., Hanna, M., et al. (2011). A framework for variation discovery and genotyping using next-generation DNA sequencing data. Nat. Genet. *43*, 491–498.

79. Van der Auwera, G.A., Carneiro, M.O., Hartl, C., Poplin, R., Del Angel, G., Levy-Moonshine, A., Jordan, T., Shakir, K., Roazen, D., Thibault, J., et al. (2013). From FastQ data to high confidence variant calls: the Genome Analysis Toolkit best practices pipeline. Curr. Protoc. Bioinformatics *43*, 11.10.1–11.10.33.

80. Gutenkunst, R.N., Hernandez, R.D., Williamson, S.H., and Bustamante, C.D. (2009). Inferring the joint demographic history of multiple populations from multidimensional SNP frequency data. PLoS Genet. *5*, e1000695.

81. Tine, M., Kuhl, H., Gagnaire, P.-A., Louro, B., Desmarais, E., Martins, R.S.T., Hecht, J., Knaust, F., Belkhir, K., Klages, S., et al. (2014). European sea bass genome and its variation provide insights into adaptation to euryhalinity and speciation. Nat. Commun. *5*, 5770.

82. De Mita, S., and Siol, M. (2012). EggLib: processing, analysis and simulation tools for population genetics and genomics. BMC Genet. *13*, 27.

83. Chen, J., Glémin, S., and Lascoux, M. (2017). Genetic diversity and the efficacy of purifying selection across plant and animal species. Mol. Biol. Evol. *34*, 1417–1428.

84. Silva, A.D., Da Silva, A., Luikart, G., Yoccoz, N.G., Cohas, A., and Allainé, D. (2005). Genetic diversity-fitness correlation revealed by microsatellite analyses in European alpine marmots (Marmota marmota). Conserv. Genet. *7*, 371–382.

85. Yashima, A.S., and Innan, H. (2017). varver: a database of microsatellite variation in vertebrates. Mol. Ecol. Resour. *17*, 824–833.

86. Roberts, L.D., Murray, A.J., Menassa, D., Ashmore, T., Nicholls, A.W., and Griffin, J.L. (2011). The contrasting roles of PPARδ and PPARγ in regulating the metabolic switch between oxidation and storage of fats in white adipose tissue. Genome Biol. *12*, R75.

87. Cochrane, G., Alako, B., Amid, C., Bower, L., Cerdeño-Tárraga, A., Cleland, I., Gibson, R., Goodgame, N., Jang, M., Kay, S., et al. (2013). Facing growth in the European Nucleotide Archive. Nucleic Acids Res. *41*, D30–D35.

88. Kent, W.J., Sugnet, C.W., Furey, T.S., Roskin, K.M., Pringle, T.H., Zahler, A.M., and Haussler, D. (2002). The human genome browser at UCSC. Genome Res. *12*, 996–1006.

89. Kent, W.J. (2002). BLAT–the BLAST-like alignment tool. Genome Res. *12*, 656–664.

STAR★METHODS

KEY RESOURCES TABLE

REAGENT or RESOURCE	SOURCE	IDENTIFIER
Biological Samples		
Reference individual (male)	this paper	Mauls 1
Re-sequenced individuals	this paper	N/A
Mauls (2 female, 1 male) Liver samples	N/A	Mauls 2-4
Gsies (2 female, 2 male) Skin/Bones samples	N/A	Gsies 1-4
LGS (2 female, 2 male) Hair samples	N/A	LGS 1-4
Deposited Data		
Genome archive	NCBI/ENA	GenBank: GCF_001458135, ENA: GCF_001458135
Genome browser	customised server	http://public-genomes-ngs.molgen.mpg.de
Experimental Models: Organisms/Strains		
Mouse male strain	Charles River Laboratories	C57Bl6 line
Rat male strain	Charles River Laboratories	Wistar line
Software and Algorithms		
DADI	bitbucket	https://bitbucket.org/gutenkunstlab/dadi
PAML	custom website	http://abacus.gene.ucl.ac.uk/software/paml.html
PSMC	github	https://github.com/lh3/psmc
DFE-alpha	custom website	http://www.sussex.ac.uk/lifesci/eyre-walkerlab/documents/dofe-31-for-linux.zip

CONTACT FOR REAGENT AND RESOURCE SHARING

Requests for further information should be directed to and will be fulfilled by the Lead Contact, Markus Ralser (markus.ralser@crick.ac.uk).

EXPERIMENTAL MODEL AND SUBJECT DETAILS

Sample collection

Four animals (two males, two females) each were obtained from three wild Alpine marmot populations in the Central Alps near Mauls (Italy, at 2367 m.a.s.l. at Mt Senges 46°52′40.55''N 11°34′56.12''E (including the reference individual), around St Martin, Gsies, (Italy) (at > 2,000 m.a.s.l, 46°49′44.2''N 12°12′15.5''E), and in the nature reserve of La Grande Sassière (at 2,340 m a.s.l., French Alps, 45°29'N, 65°90'E, animals 1426, 1442, 1467 and 1508). All animals were from different families. The animals' sex was confirmed by genome analysis (Data S1). Italian Alpine marmot samples were obtained from the Forestry and Hunting Authorities South Tyrol according to national guidelines. The fieldwork involving the French Alpine marmot samples was undertaken after deliverance of the permit number AP n82010/121 by the Préfecture de la Savoie. A.C. is authorized for experimentation with animals (diploma n8R45GRETAF110). The protocol has been approved by the ethical committee of the University of Claude Bernard Lyon 1 (n8BH2012-92 V1). All procedures involving rats and mice lipidomics analysis were carried out in accordance with UK Home Office protocols by a personal license holder.

METHOD DETAILS

General approach

To sequence, assemble and annotate a reference genome for the Alpine marmot (Figure 1A) including both sex chromosomes, we selected a wild-living male, in a typical habitat: a high altitude valley of the Central Alps that is largely free of artificial barriers due to tourism or industrial agriculture (mount Senges, near 'Mauls' village, Bolzano province, Italy, 46°52′40.5''N 11°34'56.1''E, 2367 above sea level). In order to minimize potential technology biases in low-frequency variant calling [33], genomic DNA was sequenced by two complementary sequencing technologies (Illumina and Roche/454) and different types of library protocols for illumina sequencing (Data S1). Using a hybrid assembly approach, to make the best use of short- and long-read data we assembled a genome consensus sequence of 2.51 Gbp, with a contig N50 size of ~44 Kbp, scaffold N50 size of 5.6 Mbp and superscaffold N50 size of 31.3 Mbp (Data S1). The large superscaffold N50 size was achieved by collinearity analyses based on the genome of the thirteen-lined ground squirrel

(Ictidomys tridecemlineatus, the closest relative for which a genome was available), and the house mouse (*Mus musculus*, Data S1). The draft genome assemblies of thirteen-lined ground squirrel (scaff. N50 = 8.2 Mbp) and Alpine marmot (scaff. N50 = 5.6 Mbp) were highly complementary during the collinearity scaffolding process. The Alpine marmot genome was then annotated upon the inclusion of mRNA expression data, generated by mRNA sequencing from spleen and liver tissues, employing the MAKER pipeline [34], expanded by comparative approaches as well as manual curation. Eventually, we yielded a reference set of 22,349 protein coding genes (Data S1). Of this gene set, ~19,000 genes could be annotated with gene symbols and ~14,700 associated to functional pathways (Data S1). We have refrained from attempting to include ancient DNA into the study because of the difficulties of obtaining useful samples of aDNA for the Alpine marmot, such as ancient nuclear or mitochondrial genomes. While reconstruction of ancient nuclear genomes is possible, accurate whole genome heterozygosity estimates from whole genome ancient DNA is currently very difficult to achieve. While ancient mitochondrial genomes are easier to reconstruct, their clonal, maternal inheritance, and lower effective size make them much less useful regarding the Alpine marmot's demographic past at the end of the Pleistocene.

DNA extraction, genomic sequencing and resequencing

Genomic DNA was extracted from spleen, liver, bone and hair tissues by the QIAamp DNA Mini-Kit (QIAGEN) according to the manufacturer's instructions (including proteinase K digest to obtain high molecular weight DNA). To create the Alpine marmot reference genome, we sequenced an animal from the most centrally located population (Mauls I) using Illumina Hiseq 2500 short read and Roche / 454 long read sequencing technologies. We constructed paired end (500 bp and 800 bp gel selected fragment size, Truseq version2 kit), mate pair (''gelfree'' library (MP3000) and 5kbp, 10kbp and 20kbp gel selected fragment size, Nextera Mate Pair Kit) and Roche/454 single read libraries. We produced a high sequencing coverage based on the paired end libraries and supplementary lower coverage using the matepair libraries and the 454 technology (sequencing statistics are given in Data S1). For genome resequencing of the other individuals we constructed paired end libraries with insert sizes of 300-500 bp using the Illumina Truseq version2 kit. Sequence data were generated by either Hiseq2500 (2 × 100 bp) or Nextseq500 sequencers (2 × 150 bp) (Data S1)

Assembly of a reference genome for the Alpine marmot

Prior to assembly we filtered high quality non-duplicate Illumina reads and removed adaptor sequences from the paired end reads. Mate pair reads were filtered using the Nextclip tool [35]. Next, we kept the largest region of a read that had no PhredQ value below 11 and was exceeding 32 bp in length for genome assembly. Processing of Roche/454 reads was included in the Newbler version 3 step of the genome assembly.

The sequencing reads were assembled in a hybrid approach using the IDBA assembler followed by the Newbler assembler (v3.0, similar as in [36]; IDBA version 1.1 was used to assemble all short reads into contigs and locally re-assembled contigs using iterative kmers with sizes 33,63,93,123 and 124).

Contigs and locally re-assembled contigs from IDBA were split into 29kbp fragments with 4000bp overlaps to meet maximum read length of Newbler, so that 454 data could be added to the assembly. Mate pair data were added to allow for scaffolding. We applied all filtered reads of the 5,000bp, 10,000bp and 20,000bp libraries for scaffolding. To reduce computational time of the Newbler assembly, we added only 15,000,000 read pairs of the gelfree mate pair library (MP3000) to the Newbler assembly (corresponding to ~15 X fragment or physical coverage of the genome).

The assembly short range continuity (contig N50) was improved by the GapCloser v1.12. We used Illumina libraries with high sequencing coverage in this regard (PE500, PE800 and MP3000 library). Long range continuity (scaffold to superscaffold N50) was improved by comparison with the thirteen-lined ground squirrel (*Ictidomys tridecemlineatus)* and the house mouse (*Mus musculus*) MM10 genomes. We used whole genome alignments which were done using the LAST aligner [37] to infer links by putative genome collinearity between our Alpine marmot scaffolds, which were then applied by SSPACE2 to arrange the scaffolds into superscaffolds (as described in [38]). We assigned MM10 chromosomal IDs to the superscaffolds.

Finally, we identified additional overlaps between neighboring contigs in superscaffolds by BLASTn [39] (min. identity 95%/min. length 43) and then joined these contigs (contigA end overlaps contigB start).

For genome collinearity analysis, we aligned the genome assemblies of *Marmota marmota*, *Ictidomys tridecemlineatus*, *Heterocephalus glaber* and *Mus musculus* against the assembled human chromosomes (GRCh38) using the LAST aligner [37]. Filtering for ortholog alignments was done by single_cov2. Blocks of shared collinearity were calculated by converting MAF format to the satsuma tabular format and then running the BlockDisplaySatsuma script from the Satsuma v1.17 package [40]. The BlockDisplaySatsuma script was run a second time after removal of smaller collinear blocks (< 6,000bp). The removal of these spurious blocks after round 1 resulted in larger blocks after round 2. Collinear blocks along the 22+XY human chromosomes were plotted using CIRCOS [41]. Additionally, we plotted links between collinear blocks to determine the phylogenetic position of the rearrangements.

Phylogenomic tree for rodent species

The Alpine marmot genome was aligned to whole genomes of 15 other rodent species and the human genome as outgroup. Genome assemblies were downloaded from the public NCBI assembly repository (as of January 2015). The genomes were aligned to the Alpine marmot genome using LAST [37]. The output was screened for ortholog matches using single_cov2 from the MultiZ package [42]. Pairwise alignments were combined into a multiple alignment using MultiZ. The multiple alignment file (MAF) was screened for blocks aligned in all species. All alignment blocks were concatenated into a multi fasta alignment (length 94 Mbp). We found that 500kbp fragments of the total alignment were sufficient to produce a stable tree topology using the FastTree [43] method with the

GTR model of evolution. We split the whole alignment into 188 independent segments of 500kbp and calculated trees for each segment. We compared these 188 trees to the consensus tree using CompareToBootstrap.pl from the FastTree website: http://meta.microbesonline.org/fasttree.

Spleen RNA extraction and RNASeq

For coding gene annotation RNaseq library from spleen we used QIAGEN RNAeasy kit for RNA isolation followed by the Illumina True-seq v2 RNA kit for library construction. The RNaseq libraries were sequenced by the Illumina HiSeq2500 using a paired-end protocol with read lengths of 50bp or 100bp.

Repeat annotation

In addition to repeat libraries from RepBase, Custom repeat libraries were created using RepeatModeler version open-1.0.8, RepeatScout version 1.0.5, RECON version 1.08, and Tandem Repeats Finder (TRF). RepeatMasker version 4.0.5 was used to predict repeats in the marmot genome assembly marMar2.1 from the repeat libraries.

Gene model prediction

To avoid spurious matches to the genome, low-complexity repeat regions were masked from the genome assembly marMar2.1 using RepeatMasker. The paired-end RNASeq reads were aligned to marMar2.1 using TopHat v2.0.9 [44]. The transcripts were assembled using Cufflinks and merged with Cuffmerge [45]. The predicted proteomes of human, mouse, rat and thirteen-lined ground squirrel were obtained from Ensembl [46] and UniProt [47] and the protein sequences of naked mole-rat was obtained from NCBI [48] and UniProt respectively. The gene models were predicted with MAKER [34] genome annotation pipeline in three iterations (genome browser track: "Maker"). The predicted proteomes downloaded from UniProt [47] were used for the homology search. The assembled transcripts from RNASeq reads were included as experimental evidence in the pipeline. In the first iteration, *ab initio* prediction was made using Augustus [49] with human as the training species model. In the second and final iterations, gene models that were obtained as output from the previous iteration were utilized for training SNAP [50] for *ab initio* prediction. Maximum annotation edit distance threshold of 0.75 and minimum protein size of 50 amino acids were used as thresholds for filtering of gene models.

The gene models were also predicted from a custom annotation pipeline in which gene models were predicted from homology search using SPALN aligner v2.1.2 [51], where the predicted proteins for the above mentioned species from Ensembl [46] and NCBI [48] were utilized (see genome browser track "Aligned Proteins"). We chose the best-scoring protein in a cluster based on exact exon–exon matches in a first iteration, and overlapping exons in a second iteration (track "Best Proteins match"). Second, the CDS models from SPALN were combined with spliced transcripts assembled from RNaseq using Cuffmerge [45] This resulted in a high number of possible transcript models, whose open reading frames were annotated by the Transdecoder tool (https://transdecoder.github.io). The transcript models were weighted with scores assigned for the different models based on their origin (highest rank: RNaseq only, lowest rank: SPALN only) and the open reading frames length. In addition, only gene models of at least 50 amino acids in length were retained.

The two sets of gene models were manually inspected, and the consensus gene models were chosen as the reference gene models (see track "protein coding genes"). If the gene models from the two sets were different, individual sources of evidence were utilized in choosing reference gene models.

Functional annotation

BLASTP [39] was used for Alpine marmot coding sequences against the predicted proteomes of above mentioned species obtained from Ensembl [46] and NCBI [48] databases. The functional annotation was inferred for Alpine marmot proteins from their best BLAST [39] matches. Alpine marmot proteins were also associated to gene symbols from their homologous proteins with functional annotation. The Gene Ontology (GO) terms [52] were assigned for Alpine marmot predicted proteins by identifying shared signatures with proteins of known function using InterProScan v5.17-56.0 [53]. Alpine marmot protein-coding genes were annotated to their metabolic and cellular pathways by KEGG [54] Automated Annotation Server (KAAS). This provides KEGG Orthology annotation to each gene and corresponding pathway annotations.

For orthology annotation (COG/ eggNOG, KO annotation), the predicted protein sequences were compared to the eggNOG 4.1 [55] eukaryotic database as well as KEGG [54] Release 77 database, using diamond aligner with options "blastp -k 3 -e 0.0001–sensitive." Results were post filtered using custom Perl scripts, filtering for the best hit with an alignment length of at least 50% of the reference sequence and an e-value cutoff of 1e-10. NOG categories were assigned by linking the relevant COG (http://eggnogdb.embl.de/download/eggnog_4.1/data/NOG/NOG.members.tsv.gz).

Non-coding RNA annotation

The genome assembly that was masked with RepeatMasker was also used for tRNA annotation in order to avoid spurious matches to low complexity regions. tRNA genes were annotated from the repeat masked genome with tRNAscan-SE-1.23 [56].

Mitochondrial genome annotation

Gene models for the mitochondrial genome were predicted using Open Reading Frame Finder (ORFfinder, http://www.ncbi.nlm.nih.gov/orffinder/). The functional annotations were transferred to predicted ORFs from protein coding genes of known functions from

the NCBI non-redundant sequence database [48] through BLASTP [39]. Similarly, mitochondrial tRNAs were predicted with tRNAscan-SE-1.23 [56].

Sciuridae phylogeny based on mtDNA conservation

Complete mitochondrial genomes of members of Sciuridae were downloaded from GenBank (accessed 15th Feb 2016, although excluding the genome identified as the Daurian ground squirrel (*Spermophilus dauricus* [57], because the phylogenetic placement of this genome suggests misidentification, or introgression between distantly-related species). The complete genomes were aligned with MUSCLE v. 3.8.31 [58] and manually corrected. Because highly variable regions cannot be aligned between sciurid subfamilies, we then extracted non-overlapping coding sequences, according to the annotation of Pallas' squirrel (*Callosciurus erythraeus*, GenBank: NC_025550), and made a concatenated alignment of 3,786 translatable codons. Phylogeny was estimated via maximum likelihood using RaxML v. 8.2.4 [59], using its GTR+G model and 1,000 rapid bootstraps. The phylogeny shown fits standard taxonomy [60, 61]; [62], and an identical topology was obtained when we repeated the analysis after excluding the rapidly-evolving third codon positions.

Protein coding sequence alignment across species

For all predicted marmot protein-coding genes, we obtained DNA and protein sequences of potential orthologs from nine mammals species. Seven genomes were from other rodents plus the rabbit (*Oryctolagus cuniculus*), and a human genome. Sequence annotations were obtained from the NCBI database [48] for human (*Homo sapiens*, GenBank: GCF_000001405.29), mouse (*Mus musculus domesticus*, GenBank: GCF_000001635.24), rat (*Rattus norvegicus*, GenBank: GCF_000001895.5), rabbit (GenBank: GCF_000003625.3), Upper Galilee mountains blind mole-rat (*Nannospalax galili*, GenBank: GCF_000622305.1), chinese hamster (*Cricetulus griseus*, GenBank: GCF_000419365.1), naked mole-rat (GenBank: GCF_000247695.1), thirteen-lined ground squirrel GenBank: GCF_000236235.1) and damaraland mole-rat (*Fukomys damarensis*, GenBank: GCF_000743615.1). Orthologs of the predicted Alpine marmot proteins were identified using best protein BLAST [39] hits of each refseq-annotated genome using an expect value (E) threshold of 0.01 and a minimum percent identity of 65%. Protein sequences were then aligned using MUSCLE [58]. Alignment quality at each individual position was measured using the probabilistic framework of ZORRO [63] and inconsistent positions (positional score < 9) were removed from the alignment. The filtered protein alignments were then prepared along with their respective coding DNA sequences with PAL2NAL [64] to produce codon-based alignments as input for the substitution rate analysis.

Inferring positive natural selection on protein coding genes

We used PAML [65] v4.8a to calculate the rate of substitution at nonsynonymous (amino-acid changing) and synonymous sites in protein coding genes. The ratio of these quantities is denoted $dN/dS = \omega$. Estimated ω values < 1, $= 1$, and > 1 indicate purifying selection, neutral evolution, and diversifying (positive) selection, respectively. Pairwise estimates of *dN* and *dS* of two protein coding sequences were obtained using the pairwise maximum-likelihood approach implemented in PAML (runmode = -2). We also used two branch models taking the underlying phylogeny into account. First, we tested for differences in substitution rates between the *Marmota*+*Ictidomys* clade (Figure S3A), and the remaining species using a two branch model. Second, we tested for further heterogeneity within the *Marmota*+*Ictidomys* clade, with a four branch model (Figure S3B). The two branch model was compared to a single ratio model, and the four branch model was compared to a two branch model. Significant differences between the models were assessed by likelihood-ratio tests (LRTs) which assume that $2\Delta\ln L$ is approximately $\chi 2$ distributed, with the degrees of freedom equal to the number of free parameters. *P*-values were corrected for multiple testing using the false discovery rate (FDR), according to the procedure of [66].

Gene set enrichment analysis

Genes with an FDR-adjusted *p-value* < 0.05 in 'between branches' category, and FDR-adjusted *p-value* ≥ 0.05 in 'within branch' category, were categorized as being rapidly evolving between the two clades of rodents (i.e., the clade that contains the Alpine marmot and thirteen-lined ground squirrel, and the clade that contains the other sequenced rodents). The genes that had FDR-adjusted p value ≥ 0.05 in the 'between branches' category and FDR-adjusted p value < 0.05 in the 'within branch' category were categorized as being rapidly evolving within the hibernating rodent branch, but not between the two rodent branches. In addition, other genes exhibiting rapid evolution (falling in the top 10% or 1% of *dN/dS* values) in a series of pairwise comparisons (Alpine marmot - thirteen-lined ground squirrel; Alpine marmot - human; and Alpine marmot - mouse) were also filtered for further analysis. Gene set enrichment analysis and pathway enrichment analysis was carried out on these datasets using hypergeometric testing with WebGestalt toolkit [67]. The multiple testing correction used FDR < 0.01 as the threshold for significant enrichment. In addition, gene sets involved in functions of interest, namely anti-parasite defense and fatty acid desaturation were prepared. Regardless of enrichment at pathway or gene family level, we also checked all rapidly evolving genes in "marmot - thirteen-lined ground squirrel" comparison.

Variant impact analysis between Alpine marmot and thirteen-lined ground squirrel

The marmota genome (as single_cov2 treated MAF file) was converted to sam format using maf-convert a tool which is provided with LAST aligner [37], the thirteen-lined ground squirrel genome was used as reference sequence. The sam file was converted to a bam

file, sorted and indexed using samtools [68]. We converted whole genome alignments in bam format to v*cf.* format using samtools mpileup and bcftools. The bam file was used three times as input to meet minimum coverage criteria to call SNPs and insertion/deletions (INDELs). The resulting variants were annotated using SNPeff [69] using pre-build SNPeff annotation files (spetri2.79) derived from the Ensembl [46] annotation. Genes with more than 1, 2 or 3 high impact variants were analyzed using string-db [70] (http://string-db.org). Significantly enriched KEGG [54] pathway genes for (FDR corr. P value $\leq$ 0.05) hinted at "Circadian entrainment." The corresponding genes were checked manually for signs of positive selection using the branch site analysis results described above [54, 65].

Heterozygosity analysis across species and SNP calling of Alpine marmot individuals

Complementing the Alpine marmot data, the paired-end sequence read data, genome assembly data and annotation data of other mammalian species were downloaded from their respective sources (Data S1). Reads were aligned to the genome assembly with bwa -mem v0.7.17 [71, 72]. Duplicate fragments introduced by PCR based library preparation were removed using Picard tools' MarkDuplicates (version 2.12.1-SNAPSHOT; http://broadinstitute.github.io/picard). For detecting variation in Alpine marmot samples the Genome Analysis Toolkit's (GATK version 3.6) HaplotypeCaller was used in gvcf mode [73]. Individual gvcf files were used for joint genotyping with GATK's GenotypeGVCFs tool to build a single variant file containing every Alpine marmot sample. For comparative analyses of the genic regions between marmot and other mammals the mapped read files were analyzed for variation using GATK's HaplotypeCaller (version nightly-2017-07-11) restricted to regions listed in the respective species' gff file (Data S1). Further filtering was based on base-wise coverages that were determined for these regions with bedtools coverage (v2.24.0; doi: 10.1093/bioinformatics/btq033). The "vfutils" script from SAMtools were used to further filter the SNPs. 20% of mean coverage and 200% of mean coverage were chosen as minimum and maximum coverage for variant filtering. We also required to have at least 6 supporting reads for a genotype and that heterozygous allele read are in balance, i.e., the ratio of reference allele and alternative allele is between 0.23 and 0.76 [15]. In addition, minimum RMS mapping quality (Q) of 20 was used for filtering SNPs. VCFTools v0.1.11 [74] was used for all post-filtering steps including INDEL removal, removal of homozygous SNPs and calculation of relatedness and inbreeding coefficients (–relatedness2 and –het options). Site quality value of 20 was also used as a threshold for filtering high quality SNPs. Runs of homozygosity (RoH) were calculated for each re-sequenced individual for autosomes only, using bcftools v1.7 roh [75] implemented with the -O r option, and results are shown for RoH > 2MB, which would be indicative of recent inbreeding.

Dendrogram-based Alpine marmot population analysis

SNP calling and filtering was carried out for all 12 sequenced Alpine marmot individuals as described above. Genetic distances were calculated from these matrices and cluster dendrograms were then produced from these distances. The depth of coverage of mitochondrial genomes from the 12 sequenced individuals were determined from BAM alignments using 'genomeCoverageBed' function of BEDTools [76]. The SNPs that mapped to mitochondrial genome were filtered using VCFTools [74]. A population-level variant matrix was created and the 'co-phylogenetic correlation' function was used to calculate the correlation between hierarchical clusters that were obtained from nuclear genome SNPs and mitochondrial SNPs respectively. The hierarchical clustering and co-phylogenetic correlation was carried out with *R* (v.3.4.3).

Demographic inference with PSMC

Each of the 12 Alpine marmot genomes was analyzed using pairwise sequential Markovian coalescent analysis (PSMC) [30]. Using heterozygous positions, PSMC infers rates of coalescence over time. To convert relative to absolute timescales, we assumed an average generation time, g, of 5 years [77], and a mutation rate of 2×10^{-9} per year per site. This estimate was obtained from the median divergence at synonymous sites in the nuclear genome ($ds = 0.04$) between the Alpine marmot and thirteen lined ground squirrel sequence, and assuming a split at 8.5Mya. Under the most straightforward interpretation of these plots, population sizes were much larger in the earlier Pleistocene (1myr and before), and underwent a steady decline (Figure 4E). However, this interpretation ignores the strong possibility of population subdivision, and in this case, the older events are determined by migration rate between local breeding populations, and not the species-wide effective population size [29, 30]. We therefore focused on inference of recent events.

Diffusion Approximation for Demographic Inference (DADI) and PCA

SNP calling and filtering was carried out for all 12 sequenced Alpine marmot individuals as described above. We filtered the raw SNP dataset by removing non bi-allelic and low quality SNPs (average DP < 10 or > 50, QUAL < 30). We then detected false positive SNPs (FP-SNPs) by using the two independent sequencing datasets of the reference individual. Since both datasets were from the same individual, we reasoned that any position differing by homozygous genotypes was a false positive SNP (mostly due to mapping errors in low complexity and/or duplicated regions). We thus computed the density of homozygous SNPs in 5Kb windows and removed from our dataset any window with more than 1 FP-SNP. Doing so, we discarded 96% of the detected FP-SNPs by removing 10% of the genome only. To filter out the last undetected FP-SNPs, we applied hard filters according to the GATK Best Practices recommendations [78, 79]. Hard filter values were defined by checking the distribution of the following statistics for the detected FP-SNPs: QD > 2, SOR < 3, MQ > 50, MQRankSum < −2.4, MQRankSum > 0.6, ReadPosRankSum < −2.2, ReadPosRankSum > 2.4. Finally, we masked genotypes with GQ < 10. After cleaning, 2,357,482 SNPs remained. PCA was computed with Plink v1.90b3.44 including singletons.

We then kept one SNP per 20kb-windows as a requirement for independence among loci. Such a thinning has led to a total of 178,098 SNPs left for analysis. Joint folded SFS for La Grande Sassière and Gsies populations respectively were estimated using the program $\delta a \delta i$ [80]. Thus joint SFS ranges from 0 to 4 allele counts in both samples.

We used the power of composite likelihood diffusion approximation implemented in $\delta a \delta i$ to infer demographic history of La Grande Sassière and Gsies populations. We tested a first set of 4 models including Strict Isolation (SI), Isolation with Migration (IM), Ancient Migration (AM) and Secondary Contacts (SC) [81]. In the four DADI models, an ancestral population of effective size Na splits into two daughter populations (N1 and N2, respectively) at time Ts. The two daughter populations may either not exchange migrants at all (Strict Isolation (SI), 4 parameters), or undergo continuous bidirectional gene flow (Isolation with Migration (IM), 6 parameters), or bidirectional gene flow ceasing at time Ta after the split (Ancestral Migration (AM), 7 parameters) or bidirectional gene flow starting at time Tsc after the split (Secondary Contact (SC), 7 parameters). These models were evaluated and fitted with the the observed joint SFS using 50 replicate runs per model. Models were ranked according to their log likelihoods. For nested models, comparison was performed using likelihood ratio tests. For non-nested models, we used Akaike Information Criterion (AIC) (see Table S3). Parameter estimation used a non-thinned dataset including 1,780,734 SNPs and the best-fitting model.

Coding diversity analysis

Genic diversity for coding regions was obtained for the 11 re-sequenced individuals to avoid reference bias, based on SNP calling for the $\delta a \delta i$ analysis prior to thinning, as described above. We focused on bi-allelic SNP variation and created the folded site frequency spectra for synonymous and nonsynonymous sites on a gene by gene basis using the python egglib package [82]. Statistics (θ, π and Tajima's D) were calculated on the summed site frequency spectra across all genes. Because of the evidence of population structure, we obtained population genetic statistics for each population separately as well as jointly for all 11 individuals. To estimate the distribution of fitness effects (DFE) of new nonsynonymous mutations we used a method that controls for segregation of slightly deleterious mutations [23], with the site frequency spectra for synonymous mutations as the neutral reference. Here, the strength of selection is measured by the selection coefficient s, and the efficacy of selection, by the product of the selection coefficient and the effective population size ($N_e s$). Low levels of $N_e s$ illustrate less effective (e.g., low) selection against deleterious mutations. Population genetic estimates (e.g., π_N/π_S) for populations from other animal species were obtained from [83] and [24].

Microsatellite diversity across the mammals

To compare the diversity at microsatellite loci of the Alpine marmot to other mammal species, we plotted the number of microsatellite alleles against the expected heterozygosity in a wide range of published datasets (Figure 4C). We show populations of the Alpine marmot from LGS, and estimates from other subpopulations, also from the French Alps. We included other species in the genus Marmota, such as the threatened *M. vancouverensis* and other rodent species. The Alpine marmot data come from individual published sources [18, 84], while the data from all other species were retrieved from the compilation of microsatellite data in the VarVer database [85].

Life history of the Alpine marmot in comparison to other Eutherian mammals

To compare the life history of the Alpine marmot to other Eutherian mammals (Figure 4D), we followed the approach of Bielby and coauthors [27]. These authors showed that, after correcting for body mass, much of the variance in mammalian life histories could be captured by two factors, i.e., weighted sums of multiple life history variables. One factor included contributions from neonatal mass (*g*), litter size, and gestation length (days), and can be considered as a measure of "reproductive output," in which species vary according to their investment in offspring "quality" versus "quantity." The other factor includes contributions from interbirth interval, weaning age, and age at sexual maturity (all measured in days), and can be considered as a measure of "reproductive timing," in which species vary on a "fast-slow" continuum. Figure 4D uses all records from placental mammals in the *PanTheria* database [26], which includes high quality measures of all seven quantities (the six life history variables and adult body mass). All quantities were log transformed, and then we calculated the residuals of the regressions of each variable onto body mass. We then calculated a weighted sum of these residuals using the loadings for Eutheria reported in Table 1 of reference [27].

Lipidomics

Male rats (Wistar, 6 weeks old) and male mice (C57Bl6, 6 weeks old) (Charles River Laboratories) were housed in conventional cages at room temperature with a 12-h light/dark photoperiod. All procedures were carried out in accordance with UK Home Office protocols by a personal license holder.

Lipids were extracted from 50mg of Alpine marmot, rat or mouse white adipose tissue as previously described [86]. Samples were reconstituted in 500 μL 2:1:1 isopropyl alcohol:acetonitrile:water and were analyzed in positive ion mode using a Waters Xevo G2 quadrupole time of flight (Q-ToF) mass spectrometer combined with an Ultra Performance Liquid Chromatography (UPLC) unit (Acquity, Waters Corporation, Manchester, UK). 1μl of the sample was injected onto an Acquity UPLC Charged Surface Hybrid (CSH) C18 column (1.7μm x 2.1mm x 100mm) (Waters Corporation) held at 55°C. The binary solvent system (flow rate 0.4ml/min) consisted of solvent A containing HPLC grade acetonitrile-water (60:40) with 10mM ammonium formate and solvent B consisting of LC-MS grade acetonitrile-isopropanol (10:90) and 10mM ammonium formate. The gradient started from 60% A / 40% B, reached 99% B in 18min, then returned back to the starting condition, and remained there for the next 2min. The data was collected over the mass range of m/z 105-1800 with a scan duration of 0.2 s. The source temperature was set at 120°C and nitrogen was used as the desolvation gas (900 L/h).

The voltages of the sampling cone, extraction cone and capillary were 30kV, 3.5kV and 2kV respectively, with a collision energy of 6V for each single scan, and a collision ramp from 20 to 40V for the fragmentation function. As lockmass, a solution of 2ng/l acetonitrile-water (50:50) leucine enkephaline (m/z 556.2771) with 0.1% formic acid was infused into the instrument every 30 s.

QUANTIFICATION AND STATISTICAL ANALYSIS

Statistical tests were conducted with appropriate packages in R and Python.

DATA AND SOFTWARE AVAILABILITY

The Alpine marmot genome is made available at NCBI [48] and ENA [87] genome archives (marMar2.1). The accession number for the Alpine marmot genome and sequence reads of the 11 re-sequenced individuals reported in this paper is GenBank: GCF_001458135 and ENA: GCF_001458135. For visualization, we have also made it accessible via the UCSC genome browser [88] including gene and repeat annotations, a BLAT [89] server for alignment searches and possibilities to upload and view custom data. The browser is available at http://public-genomes-ngs.molgen.mpg.de.

Current Biology, Volume *29*

Supplemental Information

Ice-Age Climate Adaptations Trap the Alpine Marmot in a State of Low Genetic Diversity

Toni I. Gossmann, Achchuthan Shanmugasundram, Stefan Börno, Ludovic Duvaux, Christophe Lemaire, Heiner Kuhl, Sven Klages, Lee D. Roberts, Sophia Schade, Johanna M. Gostner, Falk Hildebrand, Jakob Vowinckel, Coraline Bichet, Michael Mülleder, Enrica Calvani, Aleksej Zelezniak, Julian L. Griffin, Peer Bork, Dominique Allaine, Aurélie Cohas, John J. Welch, Bernd Timmermann, and Markus Ralser

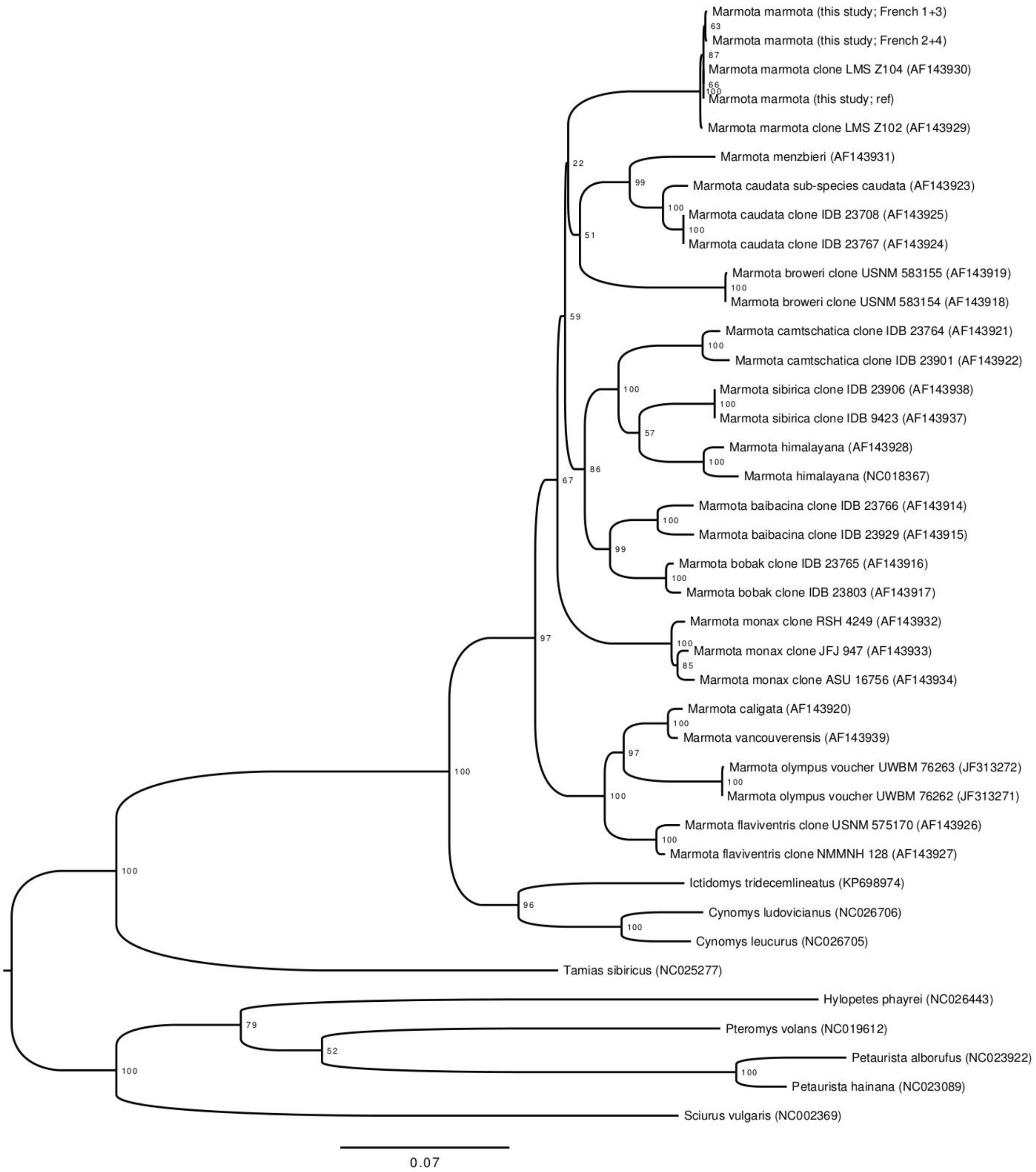

Figure S1. Phylogeny of the marmotini CytB gene confirms the phylogenetic position of the Alpine marmot. Related to Figure 1. We analysed the available sequences of Cytochrome B gene from the subfamilies Xerinae and Sciurinae, after combining these data with homologous sequences obtained from [S1]. The three distinct *CytB* sequences we sampled grouped with existing Alpine marmot specimens with 100% bootstrap support.

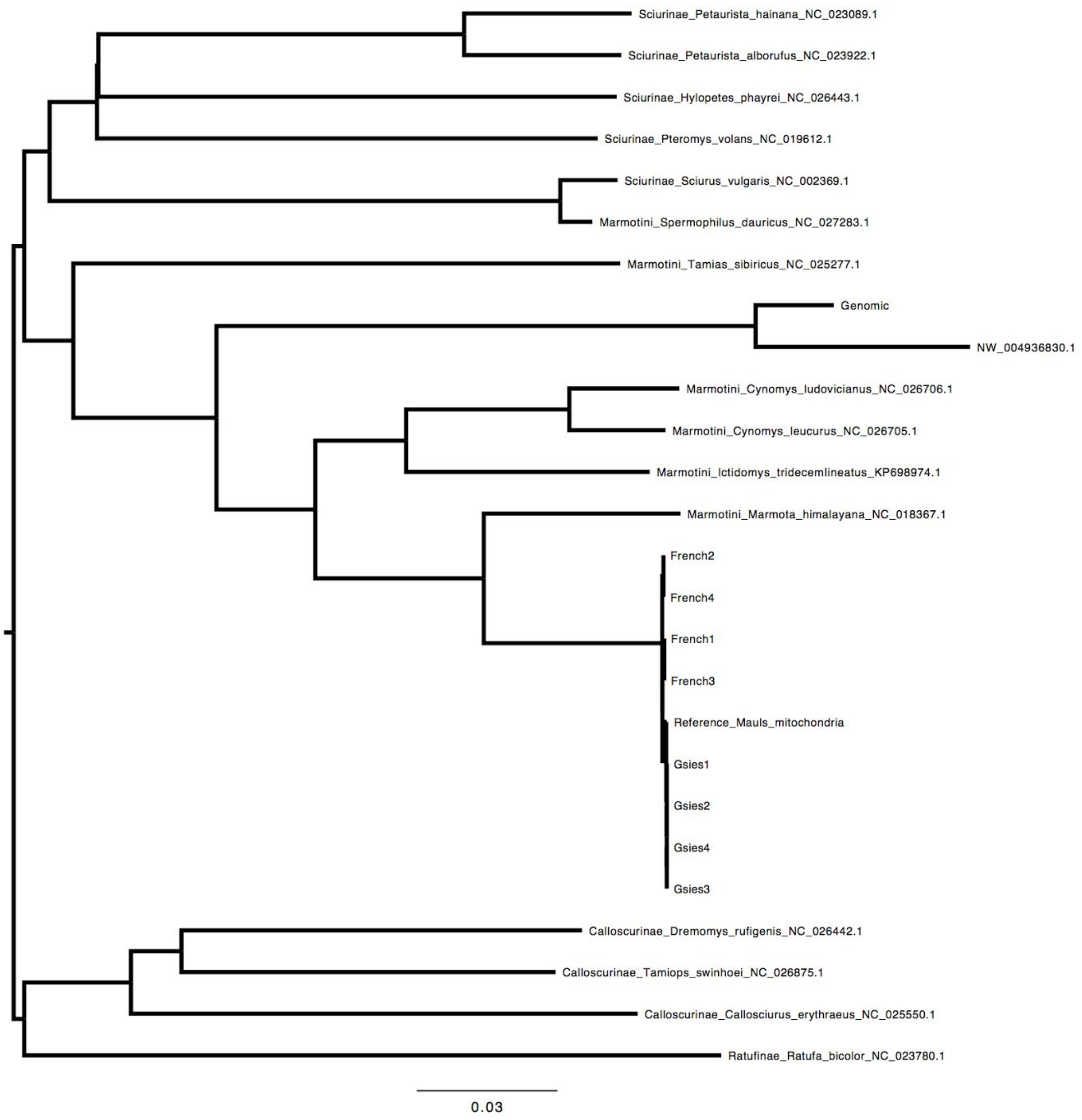

Figure S2. Phylogenetic tree based on mitochondrial genomes for multiple *Marmotini*, and sections of from the nuclear genome comprising an integration of the complete mitochondrial genome (an NUMT event). Related to Figure 1. The tree includes mitochondrial genomes of all resequenced individuals from Alpine marmots, reference mitochondrial genomes available for other *Marmotini* species in public database. The NUMT sequences from Alpine marmot is labelled '*Genomic*' in the tree, and and equivalent sequence from the nuclear genome of the thirteen-lined ground squirrel is labelled as '*NW_004936830.1*'. The placement of these sequences suggests that the NUMT was present in the common ancestor of all *Marmota*, *Ictidomys* and *Cynomys* species, but occured after their common ancestor with the Siberian chipmunk (*Tamias sibiricus*). In other words, the nuclear integration of the mitochondrial genome occurred after the divergence of *Xerinae* (Figure 1C) from other subfamilies of *Sciuridae*. The abbreviation 'French' refers to the La Grande Sassière sample 1-4.

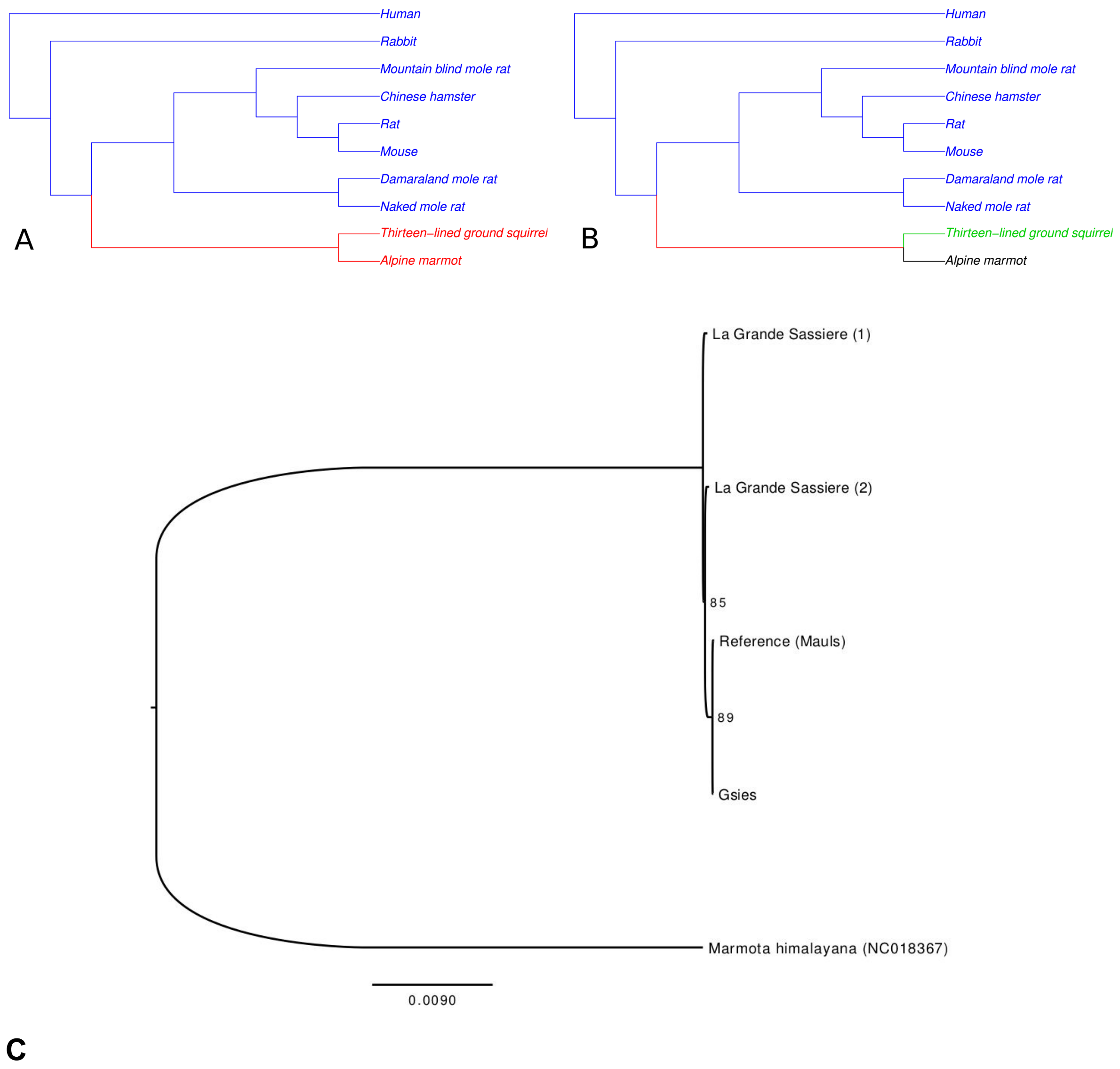

Figure S3. Underlying phylogenetic relationship of the genomes used for substitution rate analyses in PAML of protein coding genes and a genealogy from the mitochondrial genomes of our sample of sequenced Alpine marmots, rooted with the published mitochondrial genome of the Himalayan marmot (*Marmota himalayana, [S2]*). Related to Figures 2,3. Two different branch models have been used, the colours denote the branch classifications for the applied branch-site tests in PAML. (A) A two branch model and (B) a four branch model. Note, that the PAML model treats the tree as unrooted. (C) Node labels show bootstrap support, and branch lengths are shown in units of substitution per site. This genealogy supports our contention in the main text that the diversity of the two subpopulations with lower effect size (Mauls and Gsies) nests within the diversity of the subpopulation with larger effect size, from La Grande Sassiere (LGS). This is equivalent to saying that the most recent common ancestor of sequences in the LGS population is also a direct ancestor of all sequences in the sample. In particular, a grouping of the Mauls and Gsies sequences and one of the LGS sequences, to the exclusion of the other, has 85% bootstrap support. This alignment excludes any site with a gap, and support for the grouping increases to 89% when gaps are included (not shown).

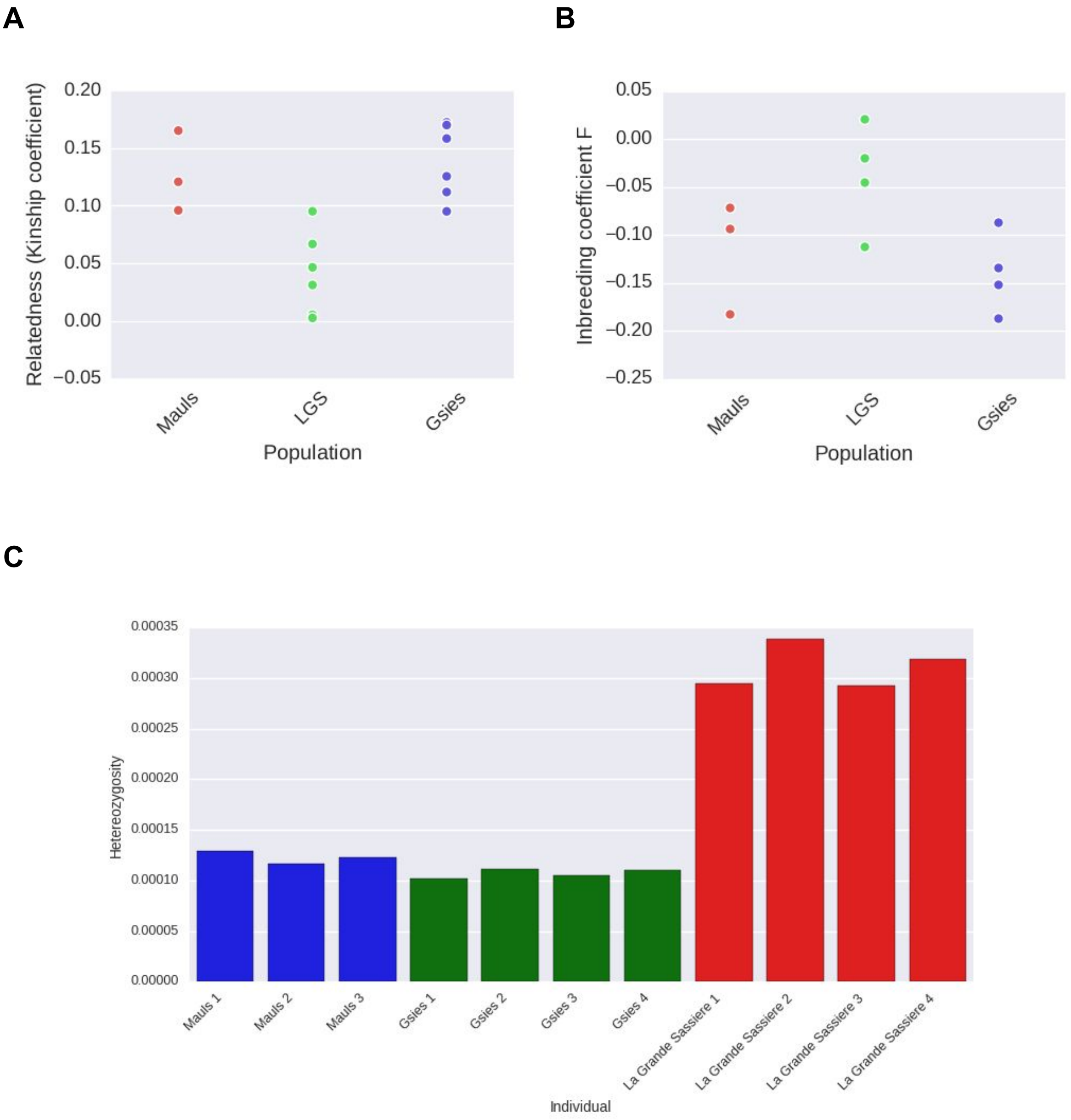

Figure S4. Population specific estimates of relatedness, inbreeding coefficients and genomic diversity. Related to Figure 3. (A) Relatedness coefficients [S3] calculated for each pair of individuals per population (3 individuals for Mauls, 4 for Gsies and 4 for LGS). A kinship coefficient of 0 denotes unrelated individuals, while a kinship coefficient of 0.5 a monozygotic twin. Individuals from Mauls and Gsies show higher levels of relatedness, similar to 2nd-degree kinship, while individuals from LGS are largely unrelated. (B) Inbreeding coefficients F, calculated for each individual within the respective subpopulation. (C) Genome-level nucleotide heterozygosity in the Alpine marmot cohorts from three different populations, Mauls (excluding the reference genome), Gsies (I), and La Grande Sassière (F). Average heterozygosity per individual is shown. Mauls and Gsies populations exhibit similar heterozygosity, whereas the animals of La Grande Sassière are around twice as diverse.

Higher in Alpine Marmot WAT		Higher in rat and mouse WAT	
**M/Z**	**ID**	**M/Z**	**ID**
738.782	DG 44:8	852.847	TG 50:0
908.913	TG 55:7	829.802	TG 50:3
900.851	TG 55:11	816.843	TG 48:4
892.883	TG 54:8	873.83	TG 53:2
896.912	TG 54:6	824.899	TG 48:0
902.954	TG 54:4	680.695	DG 39:2
864.851	TG 52:8	880.88	TG 52:0
868.874	TG 52:6	895.851	TG 55:5
894.892	TG 54:7	896.91	TG 54:6
892.876	TG 54:8	854.861	TG 51:6
862.92	TG 51:2	880.969	TG 52:0
921.869	TG 58:13	926.862	TG 56:5
872.819	TG 52:4	830.86	TG 49:4
890.953	TG 53:2	798.796	TG 47:6
925.901	TG 58:11	575.611	DG 33:4
911.893	TG 57:11		
884.905	TG 53:5		
896.821	TG 54:6		
858.888	TG 51:4		
861.827	TG 53:8		
848.817	TG 51:9		
886.921	TG 53:4		
876.936	TG 53:9		
904.97	TG 55.9		

840.838	TG 50:6		
890.868	TG 54:9		
898.923	TG 54:5		
882.89	TG 53:6		

Table S1. Enrichment of different triacylglycerol and diacylglycerol lipids, Related to Figure 2. Enrichment of different triacylglycerol and diacylglycerol lipids in Alpine marmot WATs compared to WATs of male Bl/6 mice and Wistar rats.

Individual	Number of RoH > 2MB	Percentage of genome with RoH>2MB
La Grande Sassière1	43	6.37%
La Grande Sassière2	21	2.54%
La Grande Sassière3	48	8.05%
La Grande Sassière4	30	4.14%
Gsies1	159	26.86%
Gsies2	139	21.81%
Gsies3	158	25.41%
Gsies4	147	23.05%
Mauls1	84	12.64%
Mauls2	115	15.81%
Mauls3	135	20.22%

Table S2. **Results of run of homozygosity analyses, Related to Figure 3.** Results are shown for each of the 11 resequenced marmot individuals. We measured number of long runs of homozygosity (> 2MB) as well as the proportion of the genome they covered.

Model	k	LogL	AIC	Model description	Model comparison
SI	4	-9,234.07	18,474.15	Strict isolation	
IM	6	-9,236.98	18,483.97	Isolation-with-migration	SI^{NS}
**AM**	**7**	**-6,238.64**	**12,489.29**	Ancient migration	SI***, IM***, SC+++
SC	7	-9,133.46	18,278.92	Secondary contact	SI***, IM***

Table S3. Model comparison of the migration and demography analysis using DADI, Related to Figure 4. Results of fitting four alternative models of divergence for the comparison of La Grande Sassière and Gsies populations using the SNP dataset. Models are ranked by model category. SI, strict isolation; IM, isolation-with-migration; AM, ancient migration and SC, secondary contact.

k is the number of parameters and AIC the Akaike Information Criterion. Model comparisons within and between classes of models are shown. Nested models were compared using likelihood ratio tests, with subscripts indicating significance levels (abbreviated ***P<0.001; **P <0.01; *P<0.05, NS non-significant). Non-nested models were compared using AIC with relative likelihood of each model compared to the best model L(Mi|Mbest)=exp((AICmin-AICi)/2) (abbreviated +++L(Mi|Mbest)<0.001; ++L(Mi|Mbest) <0.01; +L(Mi|Mbest)<0.05, L(Mi|Mbest)> 0.05 : AIC difference shown) [S4]

Supplemental References

S1. Steppan, S.J., Kenagy, G.J., Zawadzki, C., Robles, R., Lyapunova, E.A., and Hoffmann, R.S. (2011). Molecular data resolve placement of the Olympic marmot and estimate dates of trans-Beringian interchange. J. Mammal. *92*, 1028–1037.

S2. Chao, Q.J., Li, Y.D., Geng, X.X., Zhang, L., Dai, X., Zhang, X., Li, J., and Zhang, H.J. (2014). Complete mitochondrial genome sequence of Marmota himalayana (Rodentia: Sciuridae) and phylogenetic analysis within Rodentia. Genet. Mol. Res. *13*, 2739–2751.

S3. Manichaikul, A., Mychaleckyj, J.C., Rich, S.S., Daly, K., Sale, M., and Chen, W.-M. (2010). Robust relationship inference in genome-wide association studies. Bioinformatics *26*, 2867–2873.

S4. Christe, C., Stölting, K.N., Paris, M., Fraïsse, C., Bierne, N., and Lexer, C. (2017). Adaptive evolution and segregating load contribute to the genomic landscape of divergence in two tree species connected by episodic gene flow. Mol. Ecol. *26*, 59–76.
